# Multi-locus Analysis of Genomic Time Series Data from Experimental Evolution

**DOI:** 10.1371/journal.pgen.1005069

**Published:** 2015-04-07

**Authors:** Jonathan Terhorst, Christian Schlötterer, Yun S. Song

**Affiliations:** 1 Department of Statistics, University of California, Berkeley, Berkeley, California, United States of America; 2 Institut für Populationsgenetik, Vetmeduni Vienna, Vienna, Austria; 3 Computer Science Division, University of California, Berkeley, Berkeley, California, United States of America; 4 Department of Integrative Biology, University of California, Berkeley, Berkeley, California, United States of America; University of California Davis, UNITED STATES

## Abstract

Genomic time series data generated by evolve-and-resequence (E&R) experiments offer a powerful window into the mechanisms that drive evolution. However, standard population genetic inference procedures do not account for sampling serially over time, and new methods are needed to make full use of modern experimental evolution data. To address this problem, we develop a Gaussian process approximation to the multi-locus Wright-Fisher process with selection over a time course of tens of generations. The mean and covariance structure of the Gaussian process are obtained by computing the corresponding moments in discrete-time Wright-Fisher models conditioned on the presence of a linked selected site. This enables our method to account for the effects of linkage and selection, both along the genome and across sampled time points, in an approximate but principled manner. We first use simulated data to demonstrate the power of our method to correctly detect, locate and estimate the fitness of a selected allele from among several linked sites. We study how this power changes for different values of selection strength, initial haplotypic diversity, population size, sampling frequency, experimental duration, number of replicates, and sequencing coverage depth. In addition to providing quantitative estimates of selection parameters from experimental evolution data, our model can be used by practitioners to design E&R experiments with requisite power. We also explore how our likelihood-based approach can be used to infer other model parameters, including effective population size and recombination rate. Then, we apply our method to analyze genome-wide data from a real E&R experiment designed to study the adaptation of *D. melanogaster* to a new laboratory environment with alternating cold and hot temperatures.

## Author Summary

A growing number of experimental biologists are generating “evolve-and-resequence” (E&R) data in which the genomes of an experimental population are repeatedly sequenced over time. The resulting time series data provide important new insights into the dynamics of evolution. This type of analysis has only recently been made possible by next-generation sequencing, and new statistical procedures are required to analyze this novel data source. We present such a procedure here, and apply it to both simulated and real E&R data.

## Introduction

A common study design in population genetics consists of collecting genomic variation data from living organisms to make inferences about unobserved evolutionary and biological phenomena. The many areas where this design has been applied include demographic inference (see [1] for a recent review), recombination rate estimation [2–6], and detection of natural selection [7–13]. Recently, there has been much interest in utilizing time series genetic data—e.g., from ancient DNA [14–21], experimental evolution of a population under controlled laboratory environments [22–26], or direct measurements in fast evolving populations [27]—to enhance our ability to probe into evolution. In particular, understanding the genetic basis of adaptation to changes in the environment can be significantly facilitated by such temporal data. Specifically, the dynamics of allele frequencies in an evolving population potentially convey added information about how the genome functions [28], information which is inaccessible to methods which operate only on a static snapshot of that genome.

An experimental methodology which serially interrogates the genomes of an controlled population over time could potentially yield new insights. In fact, this methodology can now be realized thanks to the advent of next-generation sequencing. By sequencing successive generations of model organisms raised in a controlled environment, genetic time series data can be generated which describe evolution at nucleotide resolution [24, 25, 28, 29]. This so-called evolve-and-resequence (henceforth, E&R) methodology is fundamentally different than the observational approach described above, and new inference procedures are needed to analyze this type of data.

In this paper, we present such a procedure and study its ability to perform a number of testing and estimation tasks relevant to population genetics. Our method is based on an approximation to the multi-locus Wright-Fisher process, and is well-suited to the small population, discrete generation, and random mating setting in which many E&R experiments are conducted. Furthermore, because it is based on a canonical population genetic model of genome evolution, our method can directly estimate population genetic quantities such as fitness, dominance, recombination rate, and effective population size. It can also be used to design future experiments with sufficient power to reliably infer these quantities.

We first use simulated data to demonstrate the utility of our method. Then, we apply our method to analyze genome-wide data from a real E&R experiment of *D. melanogaster*, designed to study the adaptation to a novel laboratory environment over tens of generations.

### Related work

There is a small but growing literature on the analysis of evolve-and-resequence data. Feder *et al*. [30] present a statistical test for detecting selection at a single biallelic locus in time series data. (Although it is not a major focus, their method can also be used to estimate the selection parameter.) Similar to our method, they model the sample paths of the Wright-Fisher process as Gaussian perturbations around a deterministic trajectory in order to obtain a computable test statistic. However, their aim is slightly different from ours in that they analyze yeast and bacteria data sets where the population size is both large and must be estimated from data. Here we focus on population sizes which are smaller and more typical of experiments performed on higher organisms, for example mice or *Drosophila*. We generally assume that the effective population size is known but also test our ability to estimate it from data. Also, because of the increased amount of drift present in the small population regime, we necessarily restrict our attention to selection coefficients which are somewhat larger than those considered by Feder *et al*. Finally, although Feder *et al*. do study the performance of their method when time series data are corrupted by noise due to finite sampling (as in e.g. a next-generation sequencing experiment), they do not model this effect. Here we properly account for the effect of sampling by integrating over the latent space of population-level frequencies when computing the likelihood.

Another related work is Baldwin-Brown *et al*. [31], which presents a thorough study of the effects of sequencing effort, replicate count, strength of selection, and other parameters on the power to detect and localize a single selected locus segregating in a 1 Mb region. Results are obtained by simulating data under different experimental conditions and comparing the resulting distributions of allele trajectories under selection and neutrality using a modified form of *t*-test. Because it is not model-based, this method is incapable of performing parameter estimation. As a result of their study, Baldwin-Brown *et al*. present a number of design recommendations to experimenters seeking to attain a given level of power to detect selection. In a related work, Kofler and Schlötterer [32] carried out forward simulations of whole genomes to provide guidelines for designing E&R experiments to maximize the power to detect selected variants.

Illingworth *et al*. [33] derive a probabilistic model for time series data generated from large, asexually reproducing populations. The population size is sufficiently large (on the order of ∼ 10^8^) that population allele frequencies evolve quasi-deterministically. The deterministic trajectories are governed by a system of differential equations describing the effect of a selected (“driver”) mutation on nearby linked neutral (“passenger”) mutations. Randomness arises due to the finite sampling of alleles by sequencing. The main difference between the setting of Illingworth *et al*.’s and our own concerns genetic drift. While drift may be ignored when studying a large population of microorganisms, we show that it confounds our ability to detect and estimate selection in populations of order ∼ 10^3^. Thus, for E&R studies on (smaller) populations of macroscopic organisms, methods which assume that allele frequencies evolve deterministically may not perform as well as those which explicitly take drift into account.

Topa *et al*. [34] present a Bayesian model for single-locus time series data obtained by next-generation sequencing. In each time period, the allele count is modeled as a draw from a binomial distribution with number of trials equal to the depth of sequencer coverage, and success probability equaling the population-level allele frequency. The posterior allele frequency distribution is used to test for selection by comparing a neutral model to one in which unobserved allele frequencies to depend on time. In the non-neutral case, a Gaussian process is used to allow for directional selection acting on the posterior allele frequency distributions.

Finally, Lynch *et al*. [35] derive a likelihood-based method for estimating population allele frequency at a single locus in pooled sequencing data. The method allows for the possibility of sequencing errors as well as subsampling the population prior to sequencing. Using theoretical results as well as simulations, the authors give guidelines on the (subsampled) population size and coverage depth needed to reliably detect a difference in allele frequency between two populations. Unlike the other methods surveyed here, the approach of Lynch *et al*. is not designed to analyze time series data. Hence the data requirements needed to reliably detect allele frequency changes using their method—for example, sequencing coverage depth of at least 100 reads—are potentially greater than for methods are informed by a population-genetic model of genome evolution over time.

### Novelty of our method

Our method differs from the above-mentioned approaches in several regards. To the best of our knowledge, ours is the first method capable of analyzing time series data from multiple linked sites jointly. We find that this is advantageous when studying selection in E&R data. Furthermore, it enables us to analyze features of these data which cannot be studied using single-locus models, such as local levels of linkage disequilibrium and the effect of a recombination hotspot. Additionally, because our model is based on a principled approximation to the Wright-Fisher process, it can numerically estimate the selection coefficient, dominance parameter, recombination rates, and other population genetic quantities of interest. In this way it is distinct from the aforementioned simulation-based methods [31, 32], methods which only focus on testing for selection [30, 31, 34], or methods based on general statistical procedures which are not specific to population genetics [34, 35].

### Software and data availability

Source code implementing the method described in this paper is included in S1 Code. The experimental data analyzed in Analysis of a real E&R experiment data are from Franssen et al. [36] and are available on the Dryad digital repository http://dx.doi.org/10.5061/dryad.403b2.

## Results

As described above, the primary methodological advance of this paper is to derive a tractable approximation to the discrete, multi-locus Wright-Fisher model with selection. This approximation enables us to perform statistical inference on time-series data generated in E&R experiments. Before studying how our approximation performs on both simulated and real data, we give a brief overview of its motivation and derivation.

### A brief overview of the method

We consider the following model of an E&R experiment. A sexually reproducing population of *N* diploid individuals is evolved in discrete, non-overlapping generations. Pooled DNA sequencing [37, 38] is performed *T* times at generations *t*_1_ < *t*_2_ < ⋯ < *t*_*T*_. At each segregating site in the resulting data set, we assume that there are two alleles, denoted *A*_0_ and *A*_1_. (As will be seen below, up to a change in the sign of the selection coefficient associated with each site, the model is agnostic to which allele is called *A*_0_ or *A*_1_.) Let *L* and *R* denote the number of loci and the number of experimental replicates, respectively. The array **D** ∊ [0, 1]^*T*×*L*×*R*^ counts relative frequency with which the *A*_1_ allele was observed for each combination of generation, locus and replicate.

Given **D** and a vector of underlying population-genetic parameters *θ*, let ℙ(**D**∣*θ*) denote the model likelihood. In an idealized E&R experiment, generations are discrete and non-overlapping, mating is random, and the population size is fixed, so the likelihood is well approximated by the classical Wright-Fisher model of genome evolution [39]:

$$\begin{array}{c} \mathbb{P}\left(D|\theta ,G_{0}\right)=\sum_{G_{1}∊𝒢}\cdots \sum_{G_{T}∊𝒢}\mathbb{P}\left(D|G_{0},\cdots ,G_{T}\right){\mathbb{P}}_{\theta }\left(G_{T}|G_{T-1}\right)\;\cdots {\mathbb{P}}_{\theta }\left(G_{1}|G_{0}\right), \end{array}$$ (1)

where ℙ_*θ*_(*G*_*i*_∣*G*_*i*−1_) is the transition function of the discrete, many-locus Wright-Fisher Markov chain from genomic configuration *G*_*i*−1_ to *G*_*i*_ given parameters *θ*, 𝒢 is the set of all possible genotypic configurations in a diploid population of size *N*, and ℙ(**D**∣*G*_0_, …, *G*_*T*_) is the probability of the sequencer emitting **D** conditional on *G*_0_, …, *G*_*T*_. (Here, *G*_0_ represents the haplotypic configuration of the founding experimental population. In order to take advantage of linkage information we assume that this is known, although as described in Methods this is not necessary in order to use a single-locus version of our model.)

For typical problems, evaluating (1) is intractable since ∣𝒢∣ is very large and the transition density ℙ_*θ*_(*G*_*i*_∣*G*_*i*−1_) is difficult to compute and store. Asymptotic (i.e., diffusion) approximations to the transition density may be inaccurate if the population size *N* and/or scaled generation time 2*Nt* are small, as is common in an E&R experiment. Hence, alternative approximations to ℙ(**D**∣*θ*) are needed to perform inference.

The approximation we make is as follows. Let **X** ≡ (*X*_*ijk*_) ∊ [0, 1]^*T*×*L*×*R*^ denote the (unobserved) population frequency of the *A*_1_ allele at each data point. Conditional on knowing **X**, and assuming that the DNA sequencer samples each site independently and binomially from the population, we have *D*_*ijk*_ ∼ Binomial(*c*_*ijk*_,*X*_*ijk*_) where *c*_*ijk*_ is the depth of sequencing coverage observed at this site. (Although sequencer coverage is random, we assume that it is independent of all other variables in the experiment and treat it as constant conditional on the observed data.) Marginalizing over **X**, we obtain

$$\begin{array}{c} 𝓛\left(D|\theta \right)=\int_{{\left[0,1\right]}^{T\times L\times R}}\;\left[\prod_{i,j,k}𝓑\left(D_{ijk};c_{ijk},x_{ijk}\right)\right]\;{𝑝}_{X}\left(x|\theta \right)\;dx, \end{array}$$ (2)

where $𝓑(d;c,x)=\left(\begin{array}{c} c\\ d \end{array}\right)x^{d}{\left(1-x\right)}^{c-d}$ is the probability mass function of the binomial distribution and 𝑝 **X** (**x**) is the density of **X**. Note that if each *c*_*ijk*_ is large, as when the samples have been deeply sequenced, then the likelihood is (approximately) proportional to the density of **X**, i.e., 𝓛(**D**∣*θ*) ∝ 𝑝 _**X**_(**x**), and the integral in (2) does not need to be evaluated. This computational savings can be useful when performing simulations.

To perform inference we must approximate the density 𝑝 **X**, which represents the joint distribution of all allele frequencies observed in the experiment. In Methods, we provide the details of the approximation we use. Briefly, it is as follows: we assume that, conditional on the initial genome configuration *G*_0_, the underlying allele frequencies *X*_*ijk*_ are distributed according to a Gaussian process:

$$\begin{array}{c} X|G_{0},\theta ∼𝒩\left(\mu \left(G_{0},\theta \right),\Sigma \left(G_{0},\theta \right)\right) \end{array}$$ (3)

where the first- and second-order moment functions *μ*(⋅) and Σ(⋅) are obtained by considering Wright-Fisher models on a small number of loci. For example, the terms of Σ(⋅) correspond to the covariance between a pair of linked sites (potentially at different time points in the experiment) under the Wright-Fisher model. To compute this we can “marginalize out” the remaining loci in the model and study simpler Wright-Fisher model on only two loci. (A slightly more elaborate approximation is needed in the case when there is a nearby selected locus, as detailed in Methods.) Thus, we are essentially approximating the complex joint distribution of allele frequencies using a sequence of simpler one- and two-locus distributions. This approximation enables us to capture the correct mean and covariance structure in the random variable **X** while omitting higher order correlations.

Using this approximation we can perform tractable, likelihood-based inference while capturing salient aspects of the linkage-induced correlation present in the data. Indeed, by (2), (3) and the preceding discussion we have

$$\begin{array}{c} 𝓛\left(D|\theta \right)\approx \int_{{\left[0,1\right]}^{T\times L\times R}}\;\left[\prod_{i,j,k}𝓑\left(D_{ijk};c_{ijk},x_{ijk}\right)\right]\;\phi_{\theta }\left(x\right)\;dx=:\tilde{𝓛}\left(D|\theta \right), \end{array}$$ (4)

where *ϕ*_*θ*_ denotes the density function of the Gaussian distribution in equation (3). This expression may then be maximized over *θ* to perform inference. Alternatively, by placing a prior on *θ* a Bayesian approach may be adopted, but we do not explore that in this work.

### Simulated data

We tested our method on simulated data designed to capture the essential features of an E&R experiment. See Methods for the details on simulation. Briefly, it consisted of cloning a set of *F* homozygous founder lines (whose haplotypes are assumed to be known) to form an experimental population of *N* diploid organisms, which were then simulated forwards in time for *T* generations according to the Wright-Fisher random mating model. The experiment was repeated using the same starting conditions to form *R* experimental replicates. After the simulation terminated, the frequency of allele *A*_1_ was recorded for each combination of segregating site, time period and replicate, possibly with introduced sampling error; this setup mimics pooled sequencing. The input to the model consisted of these time series allele frequency data along with the haplotypes of the founder lines.

Certain aspects of the simulation were varied to test different aspects of the model; these changes are described more fully in their respective sections below. Unless otherwise noted, the simulations were performed using *F* = 200 founder lines, census population size *N* = 1000, sampling at generations *t*_*i*_ ∊ {10, 20, 30, 40, 50}, *R* = 3 experimental replicates and a region of size *L* = 10^5^ sites. These values were chosen to reflect a typical E&R experiment and we refer to them in the sequel as the “default” parameter values. Expected sequencing coverage depth is denoted by *C*, with *C* = ∞ corresponding to having perfect knowledge of the population allele frequencies. We use *C* = ∞ in the default parameter setting to upper bound the performance of our method, but also consider *C* ∊ {10, 30} to investigate the effect of uncertainty in allele frequency estimation. In these scenarios, coverage at each site was Poisson distributed with mean *C*. Lastly, scenarios with coverage “$\hat{C}$” denote simulations in which each segregating site had a random level of coverage drawn from the empirical coverage depth distribution observed in actual E&R sequencing data (see **Analysis of a real E&R experiment data** for further details.) The average coverage depth observed in this experiment was 84 short-reads, but the distribution has a heavy left tail which leads to a small percentage of sites having little to no coverage (S1 Fig).

A common objective in E&R experiments is to detect genetic adaptation. For example, a population may be partitioned, with one subgroup placed in a new environment. Upon running an E&R experiment, one wishes to 1) determine whether a fitness difference exists between the control and subject groups; 2) find the alleles responsible for the adaptation; and 3) estimate the strength of selection acting on these alleles. To test our model’s ability to perform each of these tasks, we simulated E&R experiments in which a segregating site in the founding population was chosen uniformly at random and placed under selection. The relative fitnesses of *A*_0_/*A*_0_ and *A*_1_/*A*_1_ homozygote genotypes are respectively given by 1 and 1+*s*, while the relative fitness of the heterozygote *A*_0_/*A*_1_ is 1+*hs*. In what follows, we assume *h* = 1/2 unless stated otherwise.

### Testing for selection

Let *s*_*i*_ denote the coefficient of selection at segregating site *i* = 1, …, *K*, where *K* is the total number of segregating sites in the region being considered. We wish to test the following null and alternative hypotheses:

$$\begin{array}{c} \begin{array}{c} H_{0}:s_{1}=\cdots =s_{K}=0,\\ \text{versus}\\ H_{A}:s_{j}\neq 0\;\text{for}\;\text{some}\;j, \end{array} \end{array}$$ (5)

which can be implemented using a standard likelihood-ratio (LR) test. As the number *R* of experimental replicates grows large, the distribution of the test statistic under the null hypothesis tends to a *χ* ^2^ distribution. However, since *R* was set to a realistic (i.e., small) value in our experiments, we found that the test performed better if the null distribution was determined empirically. The null distribution was calculated by performing additional simulations under neutrality (*s* = 0), computing the maximum likelihood estimate $\hat{s}$ for each simulation, and then using these estimates to compute the empirical null distribution of the LR test statistic

$$\begin{array}{c} -2\;\left[\log\tilde{𝓛}\left(D|s=0\right)-\underset{u}{\sup}\log\tilde{𝓛}\left(D|s=u\right)\right], \end{array}$$ (6)

where $\tilde{𝓛}(\text{D}|s=u)$ is defined in (4).

**Fig 1:**
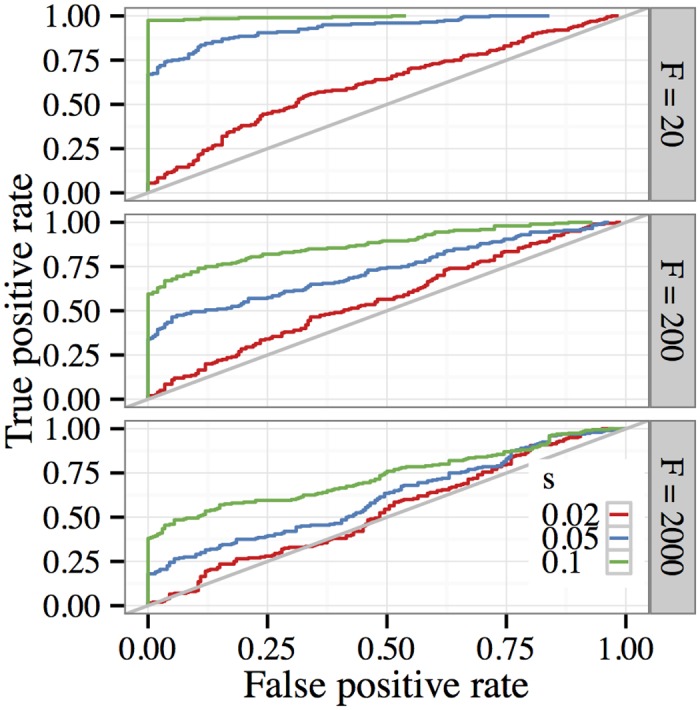
Receiver operating characteristics (ROC) when testing for selection in a region under the default parameter setting. Each ROC curve was estimated using 200 simulations. For each selection regime, the curve was calculated by comparing the distribution of the maximum likelihood-ratio over all segregating sites in a region of length 100 kb with the distribution of the same statistic under neutrality. As the plots show, stronger selection is easier to detect than weaker selection, and increased haplotypic diversity makes it more difficult to confidently reject the null hypothesis of neutrality.

Using the default parameter settings mentioned earlier, Fig. 1 displays the test’s estimated receiver operating characteristic (ROC) curve for various strengths *s* of selection and various numbers of founding haplotypes (*F*). Larger values of *F* correspond to increased haplotypic diversity in the start of the E&R experiment. Each curve was estimated from 200 simulations. Some overall trends are apparent: stronger selection is easier to detect than weaker selection, and increased haplotypic diversity makes it more difficult to confidently reject the null hypothesis of neutrality. With a small number of initial haplotypes (*F* = 20), strong selection (*s* = 0.1) is easily distinguished from neutrality. Moderate selection (*s* = 0.05) is more challenging to detect, but the test still has 75% power with a false positive rate of ∼ 6%. Weaker selection (*s* = 0.02) poses more of a challenge; in this case achieving 50% power would entail a false positive rate of approximately 30%. As the number of founding lineages increases, it becomes harder to test for selection. This occurs because many sites are segregating at low initial frequencies, increasing the chance that some are lost due to drift.

**Fig 2:**
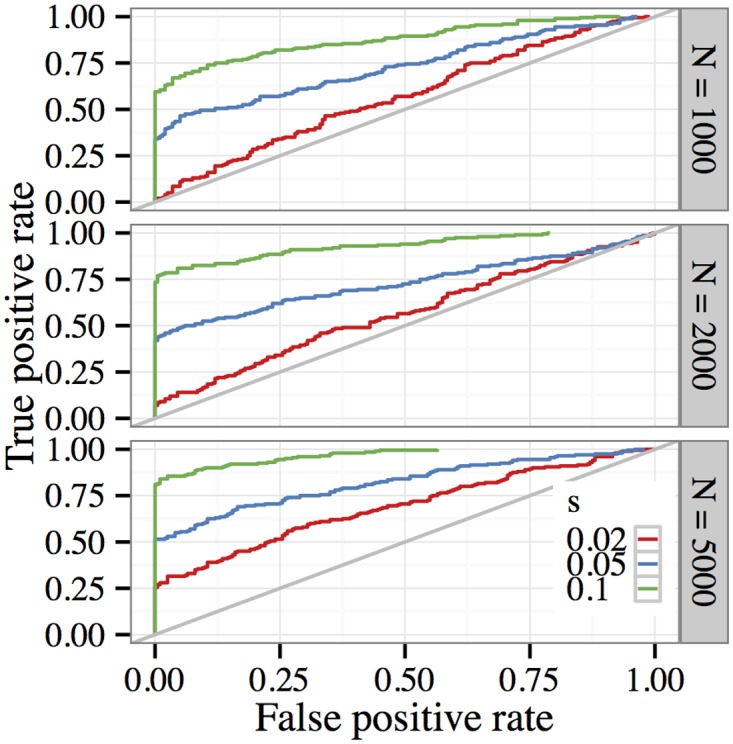
Receiver operating characteristics when testing for selection in E&R experiments with larger population sizes. Parameters for each simulation were the same as in Fig. 1, except that the population size was increased to *N* = 2000 (middle panel) and *N* = 5000 (bottom panel). Comparing these ROC curves with those in Fig. 1, we see that increasing the population size by only a few folds significantly improves the performance of the test for selection.

Detecting weakly selected variants may be confounded by genetic drift, which can cause low-frequency alleles to be lost even if they are under positive selection. One option for improving sensitivity to weaker selection is to reduce the effect of drift by increasing the effective population size used in the experiment. To study how this influences our ability to detect weaker selection, we ran additional simulations with larger population sizes *N* ∊ {2000, 5000} while holding the remaining experimental parameters fixed. Results from these experiments are shown in Fig. 2. The top panel (*N* = 1000) is reproduced from the middle panel of the preceding figure for ease of comparison. We see that reducing the amount of genetic drift in the data improves the performance of the test, particularly when it comes to distinguishing weak selection (*s* = 0.02).

### Locating the selected site

Once selection has been detected in a region, it is desirable to map the selected site as accurately as possible. An obvious estimator in this case is to declare the site with the highest likelihood-ratio (versus a neutral model) from the preceding test to be the selected site. Table 1 shows how this estimation procedure performed for different strengths of selection. We also studied how varying the number of founding lines affected the ability to precisely locate the selected site by allowing *F* to take on the values *F* ∊ {20, 200, 2000}. Since the minimum minor allele frequency (MAF) in an E&R experiment is 1/*F*, a low number of founding lines ensures that sites are segregating at intermediate frequencies, while a large value of *F* decreases LD and improves the ability to map the selected site accurately. Note that under our default parameter regime, setting *F* = 2000 amounts to sampling each founder from a panmictic population of size, so that the patterns of diversity reflect what would be seen in a (neutrally evolving) region in nature.

**Table 1:** Results of localization procedure.

		Distance					Rank					
*s*	*F*	*q*_.1_	*q*_.25_	*q*_.5_	*q*_.75_	*q*_.9_	*q*_.1_	*q*_.25_	*q*_.5_	*q*_.75_	*q*_.9_	𝔼(＃SS)
0.02	20	3540	10740	27360	51791	66270	5	17	195	660	851	1307
0.02	200	900	8870	29000	52680	74000	6	152	946	1537	1694	2086
0.02	2000	2830	10050	27380	49350	67850	14	309	1235	1889	2297	2781
0.05	20	0	0	1450	24740	50040	1	1	2	74	467	1313
0.05	200	0	0	8980	31640	52630	1	1	43	1210	1558	2086
0.05	2000	0	180	18900	43260	63470	1	2	1193	1916	2286	2778
0.10	20	0	0	0	11229	52320	1	1	1	4	14	1318
0.10	200	0	0	0	22920	48631	1	1	1	506	1566	2084
0.10	2000	0	0	8440	33841	58680	1	1	173	1955	2305	2781

The two sets of columns display percentiles of the distance in base pairs from the estimated selected site to the true selected site, and of the average rank (in terms of likelihood ratio) of the true selected site. The column labeled *q*_*j*_ corresponds to the *j*th percentile. The column labeled 𝔼(＃SS) shows the average number of segregating sites observed over all simulations. *F* denotes the number of homozygous founder lines, while *s* denotes the selection coefficient. This table shows that the selected site is generally easier to localize for larger values of *s* and *F*.

Two measures of the accuracy are displayed in Table 1. The first set of columns examines the distribution of the distance (in base pairs) between the estimated and true selected site. The second set of columns examines the distribution of the rank of the true selected site when all segregating sites in the region are sorted according to their likelihood ratio.

As the table shows, selection becomes easier to localize as it becomes stronger and as the number of founder haplotypes grows. With strong selection (*s* = 0.1) and 20 founding haplotypes, the method correctly pinpointed the exact location of the selected site in over 50% of the simulations. Additionally, the correct selected site was among the top four in 75% of the simulations. With *F* = 200 founder lines, the true selected site ranked among the top two overall in over half the simulations. The top rows of Table 1 indicate that weak selection (*s* = 0.02) is difficult to localize precisely using this method; the median estimated distance from the true selected site was 27–29 kb in these cases.

Since increasing the number *F* of founder lines diminishes linkage disequilibrium, it may seem counterintuitive that our results suggest that localizing selection actually becomes more difficult as *F* increases. In S1 Table, we have displayed the same statistics as Table 1 for the restricted subset of simulations where the selected site was segregating at an initial frequency of at least 0.1. Compared to the unrestricted data set, these sites are more likely to rise in frequency by the action of positive selection, and less likely to be lost due to drift. Here we see that increasing *F* does improve the ability to map the selected site for *s* ∊ {0.02, 0.05}; for strong selection (*s* = 0.1), essentially all cases of *F* performed equally well. Interestingly, an intermediate number of founding lineages (*F* = 200) seems to outperform both other regimes, suggesting that there is a trade-off between improving localizability by increasing *F* and limiting the number of segregating sites which must be considered by decreasing the number of founding lineages.

We also studied how coverage depth affects the ability to map the selected site. For *F* = 200, Table 2 repeats the analysis of Table 1 when the data are sampled at simulated coverage depths of 10 and 30 short-reads, as well as from the empirical coverage distribution discussed above. Comparing the two tables, we see that the additional noise introduced by sequencing makes the problem of localizing the selected site more difficult; the modal estimate is often separated from the true site by tens of kilobases. Nevertheless, in more than half the trials performed we observed that a strongly selected site would be among the top five segregating sites (in terms of likelihood ratio; see Table 2, last two rows). For medium selection, increasing coverage depth from 10 to 30 short-reads improved our ability to map the selected site by several kilobases, and more than halved the number of segregating sites we would need to examine before encountering the selected site. Weaker selection, already difficult to detect without sampling, is even more so when noise is introduced.

**Table 2:** Results of localization procedure with finite coverage.

		Distance					Rank					
*s*	*C*	*q*_.1_	*q*_.25_	*q*_.5_	*q*_.75_	*q*_.9_	*q*_.1_	*q*_.25_	*q*_.5_	*q*_.75_	*q*_.9_	𝔼(＃SS)
0.02	10	5200	15880	33670	53400	67881	34	195	795	1496	1770	2085
0.02	30	3720	11460	28850	52730	77380	25	308	912	1499	1690	2084
0.02	$\hat{C}$	5310	12419	28360	48630	60750	17	152	863	1420	1676	2082
0.05	10	0	3170	18380	42240	63170	1	6	304	1161	1635	2080
0.05	30	0	0	14330	38860	57019	1	1	129	1356	1619	2086
0.05	$\hat{C}$	990	9080	28750	53110	69750	2	4	159	1473	1639	2083
0.10	10	0	0	3770	27300	55960	1	1	5	373	1591	2082
0.10	30	0	0	290	26950	50649	1	1	2	498	1539	2091
0.10	$\hat{C}$	0	0	14079	37290	58970	1	1	3	493	1583	2082

Data were generated as in Table 1 and then sampled to simulate sequencing. The number of homozygous founder lines was fixed to *F* = 200 in this study. Average coverage depth is indicated in the column labeled *C*. The rows denoted “$\hat{C}$” correspond to simulations in which each segregating site had a random level of coverage depth drawn from the empirical coverage distribution observed in actual E&R sequencing data. The column labeled *q*_*j*_ corresponds to the *j*th percentile. The column labeled 𝔼(＃SS) shows the average number of segregating sites observed over all simulations. As the table shows, the additional noise introduced by low coverage depth makes the problem of localizing the selected site more challenging. However, under strong selection (*s* = 0.1), the true selected site was among the top five segregating sites in more than half the trials.

### Estimating the strength of selection

Once a selected site has been located, it is desirable to numerically quantify the fitness of the *A*_1_ allele. Table 3 describes the distribution of these estimates for various combinations of selective strength, coverage depth, and model complexity (i.e., the number of loci in the Gaussian process approximation). For each of the simulations above we estimated *s* by maximum likelihood. To separate the ability of our model to estimate selection from its ability to locate the selected site, we assumed that the selected site was already known when performing these estimates. Aside from varying selection strength, we also examined how coverage depth and the number of loci used for estimation affected the quality of the estimates. For each parameter combination, the table displays the mean, median and inter-quartile range (IQR) of the distribution of the maximum likelihood estimate $\hat{s}$ of *s*.

**Table 3:** Estimation of selection coefficient.

*s*	# Loci	*C*	$𝔼(\hat{s})$	Median	$\text{IQR}(\hat{s})$
0.02	1	10	0.01874	0.01957	0.02273
0.02	5	10	0.01898	0.01991	0.01862
0.02	1	30	0.01877	0.01888	0.01828
0.02	5	30	0.01988	0.01987	0.01821
0.02	1	∞	0.01724	0.01710	0.01543
0.02	5	∞	0.01775	0.01739	0.01916
0.05	1	10	0.05107	0.05047	0.02339
0.05	5	10	0.05056	0.05046	0.01775
0.05	1	30	0.05035	0.05035	0.01886
0.05	5	30	0.05072	0.05097	0.01716
0.05	1	∞	0.05018	0.04950	0.01517
0.05	5	∞	0.04840	0.04867	0.02176
0.10	1	10	0.10385	0.10255	0.02516
0.10	5	10	0.10234	0.10197	0.02204
0.10	1	30	0.10521	0.10456	0.02019
0.10	5	30	0.10265	0.10339	0.01893
0.10	1	∞	0.10403	0.10369	0.01617
0.10	5	∞	0.10300	0.10318	0.01832

For each combination of selection strength, model complexity, and coverage depth (*s*, # Loci, and *C*, respectively), the rightmost columns display the average, median and inter-quartile range (IQR) of the selection estimate $\hat{s}$ obtained from 200 simulations. Rows with *C* = ∞ denote simulations when the population-level allele frequency was known without error. When allele frequencies are sampled with noise (*C* = 10), estimates of *s* obtained from a 5-locus model generally have smaller IQR than that for a 1-locus model.

**Fig 3:**
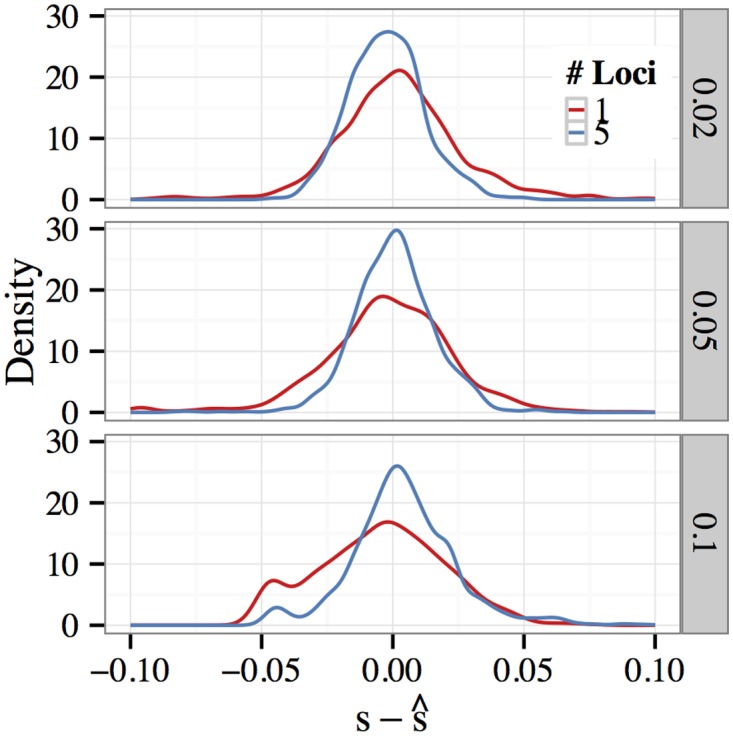
Estimated error density of with sampling. Data were generated using the standard parameters and sampled to a depth of 10 reads per site. Density estimates for the residual $s-\hat{s}$ for *s* = 0.02, 0.05, 0.10 (top to bottom) are plotted. The red and blue lines denote the density estimates obtained using one- and five-locus models, respectively. The five-locus model, which takes additional data from linked sites into account, produces estimates which are more concentrated around the true parameter value.

Several interesting features emerge from the table. Inter-quartile range is of roughly the same order across scenarios, so that estimation error shrinks relatively as selection become stronger. For one-locus models, IQR shrinks as coverage depth increases. For multi-locus models the effect of increasing the number of sites used to perform estimation is interesting. When the data are observed without noise, we saw little improvement in the accuracy of $\hat{s}$ when using a single-locus model fit only to data from the selected site versus a multi-locus model which also took the trajectories of linked sites into account. In fact, in several cases this cause the estimates to become more dispersed as the trajectory of the selected allele had relatively less weight in the likelihood calculation. On the other hand, when allele frequencies are sampled with noise we see that estimates $\hat{s}$ obtained from a five-locus model generally have smaller IQR, particularly in the low-coverage-depth case *C* = 10. These findings are confirmed in Fig. 3, which displays density estimates for the residual $s-\hat{s}$ for each of these cases presented in the table. Compared with the one-locus model, the five-locus model which takes additional data from linked sites into account produces estimates which are more concentrated around the true parameter value. Thus, when the data are noisy (i.e., when *C* is small), the trajectories of nearby linked sites provide useful information concerning the (unobserved) population frequency of the selected allele as it evolves over time.

We observed a slight negative bias for weaker selection and a slight positive bias for medium and strong selection, which can be attributed to loss or fixation of the selected allele. Indeed, estimated selection may be negative when a weakly selected allele segregating at low frequency is lost due to drift; similarly, there is a tendency to overestimate the strength of selection acting on a high-frequency allele which fixes quickly.

It is also interesting to consider the effect of study design on estimation accuracy. In Table 4 we examine how parameter estimates are affected by sequencing effort and experimental duration. We focus on the limited-coverage case (*C* = 10) since it is most sensitive to adding or removing sequence data from additional generations. For ease of comparison, the first set of rows reproduces data from Table 4, where generations {10, 20, 30, 40, 50} were sequenced. The next subsection examines the case when sequencing effort is reduced to two time periods {25, 50}. The final subsection studies estimation quality when the experimental duration is halved, and only one round of sequencing is performed at generation 25. In all cases we see that the estimators are approximately unbiased, $𝔼(\hat{s})\approx s$, but that their dispersion about the true parameter value is greatly affected by data availability. Sampling genomic data at just a single time period *t* = 25 roughly doubles the IQR of the estimator in each case. Interestingly, with two time periods (*t* ∊ {25, 50}) performance is improved, and the estimator is only somewhat less precise than when sampling at every tenth generation. Finally, as in the previous table we see again that, at least for data sampled at low coverage, estimation performance is unilaterally improved by fitting a multi-locus model versus a single-locus model.

**Table 4:** Effect of sampling frequency on selection coefficient estimation.

*s*	# Loci	*C*	$𝔼(\hat{s})\approx s$	Median	$\text{IQR}(\hat{s})\approx s$
			*t*_*i*_ ∊ {10, 20, 30, 40, 50}		
0.02	1	10	0.01874	0.01957	0.02273
0.02	5	10	0.01898	0.01991	0.01862
0.05	1	10	0.05107	0.05047	0.02339
0.05	5	10	0.05056	0.05046	0.01775
0.10	1	10	0.10385	0.10255	0.02516
0.10	5	10	0.10234	0.10197	0.02204
			*t*_*i*_ ∊ {25}		
0.02	1	10	0.01742	0.02231	0.05067
0.02	5	10	0.01938	0.02086	0.03450
0.05	1	10	0.04958	0.04813	0.05762
0.05	5	10	0.04864	0.04887	0.03045
0.10	1	10	0.09913	0.10167	0.05164
0.10	5	10	0.09930	0.09948	0.03535
			*t*_*i*_ ∊ {25, 50}		
0.02	1	10	0.01912	0.01886	0.02799
0.02	5	10	0.01948	0.01953	0.01923
0.05	1	10	0.05149	0.05047	0.02591
0.05	5	10	0.05142	0.05037	0.01969
0.10	1	10	0.10360	0.10256	0.03049
0.10	5	10	0.10139	0.10105	0.02208

Column definitions are the same as in Table 3. The three sections correspond to sampling at generations (10, 20, 30, 40, 50), 25, and (25, 50) respectively. The estimators are approximately unbiased in all cases, but their dispersion about the true parameter value is considerably affected by data availability. Further, the 5-locus model consistently produced improved estimation results over the 1-locus model.

### Overdominance estimation

In the preceding discussion, the dominance parameter was fixed at *h* = 1/2, so that selection acted additively. Our method is capable of handling general diploid selection. In our experiment, we tested our method’s ability to estimate the effect of overdominance, in which case heterozygotes are fitter than either homozygote. We simulated populations under the conditions *h* > 1 and *s* ≪ 1 such that heterozygotes had a relative fitness of 1+*hs* where *hs* ∊ {0.02, 0.05, 0.10}. Thus, heterozygotes have a fitness advantage of the same order as that which we were able to detect in the additive case.

Results for jointly estimating *h* and *s* are shown in Table 5. A fixed value of *s* = 0.01 was used for fitness in all cases, while *h* was varied. We found that estimating overdominance is difficult when both alleles are initially segregating near their limiting frequency of ½, since the resulting allele trajectories appear very similar to those generated by a neutral model with drift. The results in the table are therefore conditioned on the initial allele frequency residing outside of the interval [0.4, 0.6].

**Table 5:** Overdominance estimation.

*h*	*hs*	$𝔼(\hat{s})$	$\text{IQR}(\hat{s})$	$𝔼(\hat{h})$	$\text{IQR}(\hat{s})$	$𝔼(\hat{h}\hat{s})$	$\text{IQR}(\hat{h}\hat{s})$
2.0	0.02	0.023	0.018	3.28	4.92	0.029	0.016
5.0	0.05	0.012	0.009	4.60	11.19	0.048	0.022
10.0	0.10	0.010	0.005	6.62	7.28	0.099	0.024

The selection coefficient was fixed at *s* = 0.01 while the dominance parameter *h* was varied. In each simulation, the initial allelic frequency was restricted to lie outside the interval [0.4, 0.6] (see discussion in text). The estimators $\hat{h}$ and $\hat{s}$ are highly variable, while the product estimator $\hat{h}\cdot \hat{s}$ is substantially more accurate.

**Fig 4:**
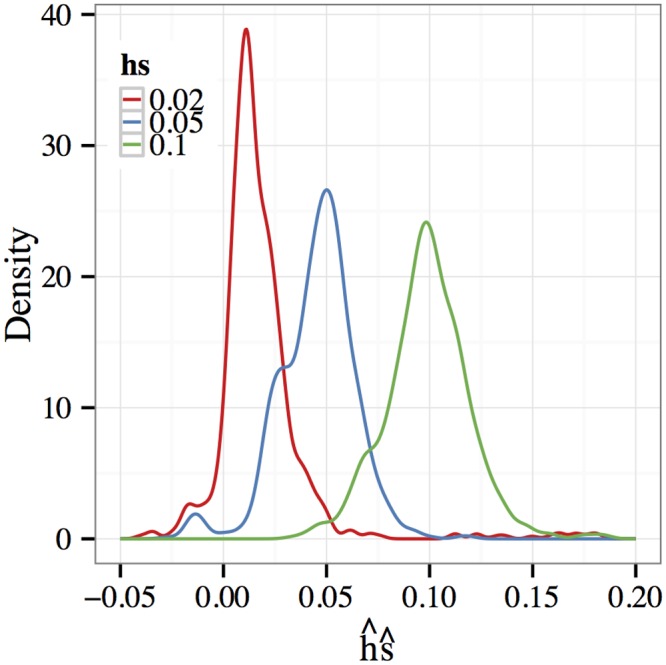
Overdominance estimation. Density estimates of the product $\hat{h}\cdot \hat{s}$ when the parameters are estimated jointly. The selection coefficient was fixed at *s* = 0.01 while the dominance parameter *h* was varied. In each simulation, the initial allelic frequency was restricted to lie outside the interval [0.4, 0.6] (see discussion in text). The mean of $\hat{h}\cdot \hat{s}$ is quite close to the true value *hs* and the distribution is tightly concentrated around that value.

When considered individually, the estimators $\hat{h}$ and $\hat{s}$ are highly variable (see Table 5, columns 3–6). This behavior is expected since, as witnessed in the previous subsections, small values in *s* (specifically, *s* = 0.01) are difficult to detect in experimental data. Encouragingly, a different picture emerges when we consider the product estimator $\hat{h}\cdot \hat{s}$ (see Table 5, columns 7–8). The estimator is close in expectation to the true value *hs* (column 2) and also more tightly concentrated around that value. Density estimates of the product estimator $\hat{h}\hat{s}$ are shown in Fig. 4 and confirm this finding. Each density estimate has a mode at the true parameter value *hs* and is reasonably concentrated around that value.

### Recombination rate estimation

**Fig 5:**
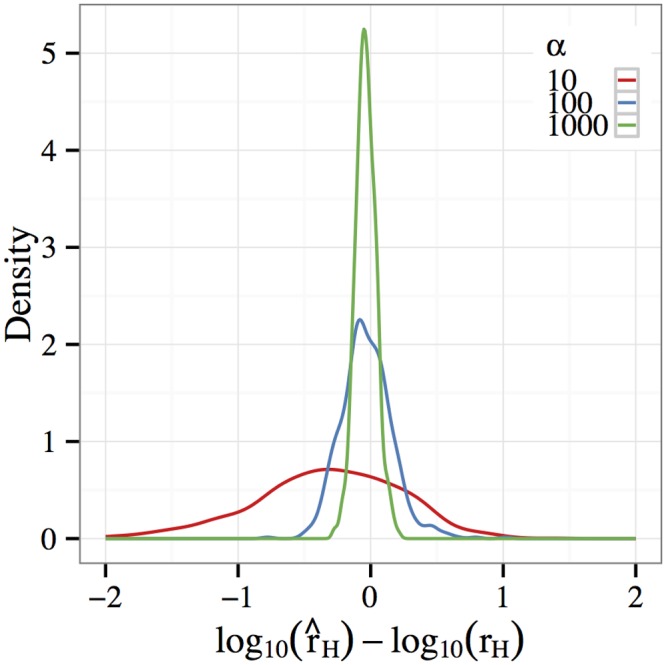
Hotspot estimation. A recombination hotspot was simulated by evolving a 100 kb region in which the recombination rate *r*_*H*_ = *α* ⋅ *r* for the middle 2 kb (positions 49–51 kb) was increased by a multiplicative factor *α* ∊ {10, 100, 1000} above the baseline recombination rate *r*. The hotspot intensity ${\hat{r}}_{H}$ was then estimated from E&R experimental data. The figure shows density estimates of the residual $\log_{10}({\hat{r}}_{H})-\log_{10}(r_{H})$ for each value of *α*. Note that the mode of the density is close to zero in all cases. Furthermore, a 3-order increase in *r* is easily detected, while a 2-order increase can also be estimated to well within an order of magnitude of accuracy.

Our multi-locus model can also be used to study phenomena which alter covariance between linked alleles. For example, in a region containing a recombination hotspot, covariance decreases markedly as increased recombination breaks down linkage disequilibrium. Using the same likelihood-based approach as above, the recombination rate within the hotspot can be estimated from E&R data. To test this, we simulated a region of length *L* = 100 kb in which the middle 2 kb region had an elevated recombination rate *r*_*H*_ = *α* ⋅ *r*, where *r* = 10^−8^ is the background recombination rate and *α* ∊ {10, 10^2^, 10^3^}. For simplicity, we focused on the case of *C* = ∞ and assumed that the hotspot boundaries are known. For each simulation, a 30-locus model was fit using 10 randomly-selected loci from within the hotspot and 20 outside of it. Density estimates for the residual $\log_{10}({\hat{r}}_{H})-\log_{10}(r_{H})$ are shown in Fig. 5. In all cases, the mode of the density occurs close to zero. A 3-order increase in the recombination rate is easily detected in experimental data, and a 2-order increase can also be estimated to well within an order of magnitude of accuracy. Increasing the recombination rate by only a factor of 10 leads to a fairly dispersed estimator, and it would be difficult to detect using the default experimental parameters.

### Effective population size estimation

**Fig 6:**
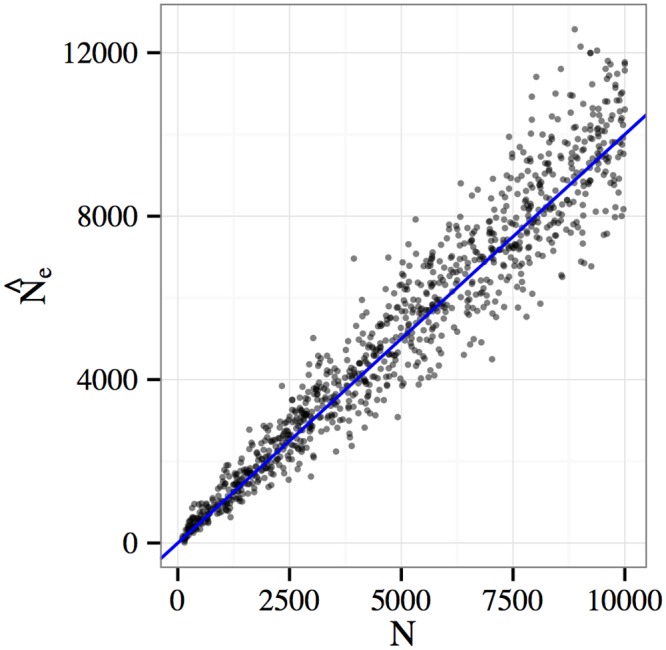
Effective population size estimation. The census population size (*N*) versus the estimated effective population size $\left({\hat{N}}_{e}\right)$ for 1,000 simulated E&R experiments. For each simulation, population size was chosen uniformly at random from the interval [10, 10^4^]. The estimator is quite accurate for small *N*, but becomes more variable as *N* grows. See text for discussion.

As a final application of our method, we consider estimating the effective population size *N*_*e*_ from experimental data. Up to now we have assumed that the (census) size *N* of the experimental population is fixed at a known value. In practice, the effective and census population sizes may differ due to various factors, including nonrandom mating and population structure. It could be interesting to quantify this effect by estimating *N*_*e*_ in experimental data using the same likelihood-based procedures described above. Since our model approximates the Wright-Fisher process, in which *N*_*e*_ = *N*, and simulations were carried out also assuming the Wright-Fisher model, we expect our estimate ${\hat{N}}_{e}$ to be close to *N*. Fig. 6 shows a scatter plot of ${\hat{N}}_{e}$ versus *N* for 1,000 simulated E&R experiments. In each experiment, the population size *N* was chosen uniformly at random from the interval [10, 10^4^]. We see that the estimator is quite accurate for small population sizes and becomes more variable as *N* grows. This is expected since ${\hat{N}}_{e}$ is essentially measuring genetic drift, which is of order *O*(1/*N*) as *N* grows. Thus, the inverse map taking drift to population size is well-conditioned for small *N* and becomes ill-conditioned as *N* grows.

### Analysis of a real E&R experiment data

Finally, we tested our method on data from an actual E&R experiment of *D. melanogaster* adapting to a new laboratory environment involving an alternating cycle with 12-hrs of cold (18^∘^C) and 12-hrs of hot (28^∘^C) temperature conditions. The experiment has been described previously [25, 36], so we give only a brief summary here. The experiment consists of three *D. melanogaster* populations each of *N* ≈ 1000 individuals. The populations were founded by gravid females from isofemale lines, and then evolved forward in discrete generations. Pooled sequencing was performed at generations 15, 37, and 59 on three experimental replicates.

The observed coverage distribution for a selected data point (replicate 4, generation 59) is shown in S1 Fig. The distribution has fairly high average coverage depth, but a significant number of sites have little or no coverage. After read-mapping and filtering sites to have sufficient coverage and quality, 1.46 million segregating sites remained in the data set. In order to maximize the accuracy of our model, we further filtered the data to include sites segregating only at intermediate frequencies (MAF ≥ 0.1), resulting in a total of 414, 049 sites. The distribution of coverage for each filtered pool-seq data point is plotted in S2 Fig. In addition to pooled sequencing data, whole-genome haplotype sequences were collected for 29 founder individuals (see [36] for details). This enabled us to estimate local linkage disequilibrium for use in the multi-locus model.

We employed a two-pass approach to analyze the data. In the first pass, we performed a genome-wide scan of the entire data set using the single-locus implementation of our model. Using the results of this first pass, we identified regions of the genome for which there was strong evidence of non-neutrality. We then fit more computationally demanding 3-, 5-, and 7-locus models in these genomic regions in order to localize and estimate the strength of selection. Further details of our analysis procedure are provided in Methods. Total run-time for the one-locus portion of the analysis was 8 hours 43 minutes for the entire genome (≈ 0.07 seconds per site), using a parallel implementation on a 16-core machine. For the multi-locus models, the average running time per site was 0.94 seconds (3 loci), 2.54 seconds (5 loci) and 4.96 seconds (7 loci). Memory consumption for the multi-locus models averaged around 40 GB, although this can be reduced at the expense of greater run-time by disabling result caching features built into our software.

**Fig 7:**
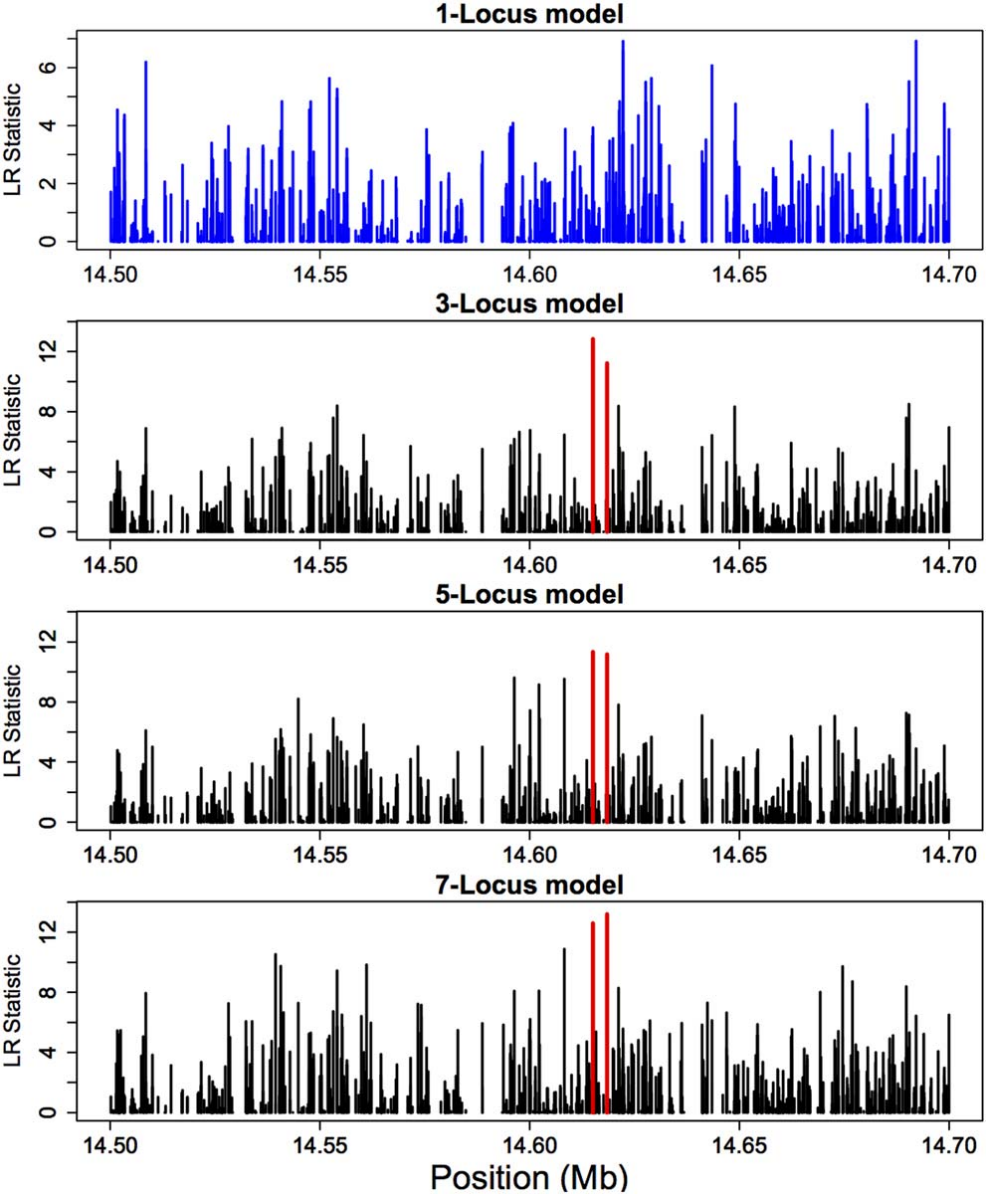
Comparison of likelihood-ratio results for the one-locus and multi-locus models applied to a real E&R experiment of *D. melanogaster*. Shown here are the results for a 200 kb region of chromosome arm 3R. Note that the one-locus model resulted in several distant SNPs with comparably high LR values, while all multi-locus models produced cleaner pictures, isolating two nearby SNPs (illustrated in red) in the 14.615–14.619 Mb region with pronounced peaks.

The first pass identified the following 16 intervals (in Mb) for further analysis: Chr X: (1.6, 1.7); Chr 2L: (15.0, 16.0), (16.5, 18.5), (19.0, 20.7); Chr 2R: (20.9, 21.1); Chr 3L: (2.3, 3.0), (6.6; 6.7), (8.6, 8.8), (13.0, 14.5), (15.2, 16.0), (18.0, 18.9), (20.2, 20.8); Chr 3R: (14.3, 14.7), (15.7, 16.1),(18.4, 19.0), (26.2, 26.4). Focusing on these regions, we computed the LR test statistic at about 37,000 SNPs in total for each multi-locus model. Because of long-range linkage disequilibrium and hitchhiking effects [36], all models produced rather large LR statistics for numerous sites. However, compared to the one-locus model, multi-locus models generally produced more distinctive peaks in the LR statistic. For example, Fig. 7 illustrates a 200 kb region of chromosome arm 3R for which the one-locus analysis resulted in several distant SNPs with comparably high LR values, while all multi-locus models highlighted two nearby SNPs (illustrated in red) in the 14.615–14.619 Mb region with pronounced LR peaks. S3 Fig is another example of size 800 kb for which every multi-locus model yielded a distinctive peak (shown in red) near 18.205 Mb of chromosome arm 3L, while the one-locus model did not single out any particular SNPs in the region.

To deal with variable results across different multi-locus models, we used the following strategy: For each of 3-, 5-, and 7-locus models, we first ranked the SNPs according to their LR statistic and took the top 100 SNPs. This corresponds to the LR statistic being greater than 8.741, 9.525, and 11.310 for the 3-, 5-, and 7-locus model, respectively. (Shown in S4 Fig are empirical cumulative distributions of the LR statistic for each multi-locus model; the 99th percentile for the 3-, 5-, and 7-locus models are 6.883, 7.330, and 8.257, respectively.) Then, we took the intersection of the resulting three top 100 lists. This led to thirteen SNPs, nine of which belong to five coding genes (one SNP in CG42334 and two SNPs each in CG9726, CG33991, CG17697, and CG7720). In particular, gene CG7720 actually resides in the region illustrated in Fig. 7, and the two distinctive SNPs mentioned in the previous paragraph are in fact the two top ranking SNPs contained in CG7720. Allele frequency trajectories of the thirteen identified SNPs are illustrated in S5 Fig; they generally display an increasing trend over the time course of the experiment. A brief description of the five genes is provided in Table 6. It is well known that temperature affects the cell membrane composition [40], and it is interesting that one of the five genes we identified is involved in transmembrane transport. It is also interesting that two of the remaining genes are related to cytoskeleton (reorganization and coordination).

**Table 6:** Genes identified by our analysis as potentially being under selection in the E&R experiment [25, 36] of *D. melanogaster*.

Gene	Chr	Position	Biological process
CG33991 (nuf)	3L	14, 183, 976–14, 225, 600	Cytoskeleton reorganization; microtubule-based process; wing disc dorsal/ventral pattern formation
CG17697 (fz)	3L	14, 267, 446–14, 326, 917	Receptor for Wnt proteins; establishment or maintenance of cell polarity; G-protein coupled receptor signaling pathway; required to coordinate the cytoskeletons of epidermal cells to produce a parallel array of cuticular hairs and bristles
CG42334 (comm3)	3L	15, 606, 283–15, 640, 837	Autophagic cell death
CG7720	3R	14, 584, 955–14, 620, 876	Transmembrane transport
CG9726 (PH4alphaMP)	3R	26, 317, 530–26, 321, 872	Peptidyl-proline hydroxylation to 4-hydroxy-L-proline; oxidation-reduction process

Genomic coordinates correspond to that of BDGP Release 5 assembly, and biological functions are taken from FlyBase.org.

Using the same data, Franssen *et al*. [36] recently studied the evolving pattern of linkage disequilibrium and identified 17 haplotype-blocks putatively under selection. Interestingly, three of the five genes mentioned above—namely, CG33991, CG17697, CG7720—are contained in that set of haplotype-blocks.

## Discussion

In this paper we have presented a model for analyzing time series data generated by evolve-and-resequence experiments. Our model is designed to analyze multiple recombining sites evolving in a moderately-sized population and potentially affected by measurement error. On data obtained from simulated E&R experiments combined with pooled sequencing, we have shown that it is possible to detect, localize and estimate the strength of selection in the range *s* ∊ [0.01, 0.10] in a population of moderate size (*N* ∼ 10^3^) and using a moderate number (*R* = 3) of experimental replicates. We have also explored the effect of the founding population composition (in terms of the number of founders) and sequencer effort (coverage depth, number of sampling time points, and time intervals between sampling) on the quality of these estimates. Finally, we have shown that our method can also be applied to study other phenomena of interest, including overdominance and effective population size; in particular, our work suggests that E&R data can be used to estimate recombination rates in putative hotspots in model organisms inferred by previous studies [5, 41, 42]. Space and time considerations have necessarily prevented us from considering many other combinations of experimental parameters which could be informative when designing E&R experiments. To enable other researchers to explore these options, we have made the computer code used in this study publicly available.

We have also applied our method to analyze genome-wide data from a real E&R experiment of *D. melanogaster* adapting to a new laboratory environment over tens of generations. Because of the small population size involved in that particular E&R experiment, LD does not break down fast enough over the time scale of the experiment, and long-range correlation between distant sites and hitchhiking effects pose challenges to localizing the true sites under selection. In our work, we have observed that combining information from several multi-locus models may produce improved results. We have employed a heuristic ensemble approach in this paper; further statistical work on this problem would be worthwhile to pursue in the future. In a given multi-locus model, we have noticed that choosing appropriate SNPs to include in the model is important for producing cleaner signals. Specifically, we recommend choosing SNPs for which the allele frequency does not get too close to the boundary (0 or 1) and that are sufficiently far apart (e.g., > 100 kb apart for the particular E&R data we considered). Our analysis of the E&R data has identified five genes in *D. melanogaster* (Table 6) which may be involved in adaptation, and some of these genes reside in haplotype-blocks recently identified as candidate regions of selection [36]. Further, some of the genes we have identified are involved in related biological processes, in particular concerning cytoskeleton and transmembrane transport. It would be interesting to investigate this thread of observations further. We note that we have employed a rather conservative approach in our analysis, so it is likely that we missed several other regions potentially under selection.

Experience has shown that the running time of our model is dominated by the recursive procedure used to calculate covariances between pairs of sites (see Methods). Thus, to fit a *K*-locus model sampled at *T* time points has computational complexity of order *O*(*K* ^2^ *T* ^2^). When performing the large number of simulations needed to benchmark our model, this quadratic scaling in the model size *K* prevented us from fitting models jointly using many more sites. Since our results suggest that estimation precision can be improved (in particular, at low coverage) by exploiting linkage information between sites, it could make sense in practice to expend additional computation time in order to add more sites into the model.

It is interesting to compare our findings with existing results. Feder *et al*. [30] suggest that power to detect selection is maximized when (positively) selected alleles are sampled as they rise in frequency, but before they have fixed. By a simple modification of their argument, the expected strength of selection required for a mutation in our simulated E&R experiments to achieve frequency *x*_*f*_ in *T* time periods is given by

$$\begin{array}{c} s_{\text{fix}}\left(T\right)=\frac{1}{H_{F-1}}\sum_{k=1}^{F-1}\frac{1}{kT}\log\left(\frac{x_{f}}{1-x_{f}}\cdot \frac{F-k}{k}\right), \end{array}$$ (7)

where $H_{n}:=\sum_{i=1}^{n}1/i$ is the harmonic series. Above we generally chose *T* = 50 and *F* = 200; for *x*_*f*_ = 0.95 we find that *s*_fix_(*T*) ≈ 0.11 which roughly agrees with our finding (Fig. 1) that medium and strong selection (*s* = 0.1) could be reliably detected, while weaker selection was fairly difficult to detect. Our findings are somewhat more optimistic than those of Baldwin-Brown *et al*. [31], whose simulation results suggest that E&R experiments require a fairly large number of experimental replicates (*R* ≥ 25), founder haplotypes (*F* ≥ 500) and strong selection (*s* ≥ 0.1) in order to reliably detect and localize selected sites in a 1 Mb region. Since we used a smaller region for simulation (*L* = 100 kb), the results we report are not directly comparable; nevertheless, it is interesting that with many fewer replicates and haplotypes (*R* = 3 and *F* = 20) we could reliably detect the selected site in at least 50% of trials (Table 1). With sampled data the problem becomes harder, but we found that average coverage depth 30 still sufficed to discover the selected site from among the top four segregating sites in 50% of trials (Table 3).

Several extensions to our model could potentially be of use. In our simulations we assumed that sequencer coverage depth is Poisson distributed. However, some studies have noted that coverage depth is overdispersed relative to the Poisson distribution, in which case an alternative distribution such as the negative binomial is preferred. For multi-locus estimation problems, our model requires that the haplotypic structure of the founding experimental population be known. In cases where this information is not known exactly, a Bayesian approach could be adopted in which model results are weighted by a prior on the space of initial haplotypic configurations. Such a procedure could allow the researcher to trade sequencing effort for computation time by decreasing the burden of initial sequencing that must be performed in order to establish the haplotypes of the founding lineages.

The other extreme of sequencing effort is to obtain haplotype data for a sample of individuals at each sampling generation, rather than to use pooled sequencing to infer only marginal allele frequencies. (Indeed, there is a discussion on the utility and power of pooled sequencing [37, 43–45].) The same multi-locus model underlying our approach can be applied to develop a method for analyzing haplotypic time series data, and we will explore incorporating such an extension into our method.

Our approximation to the multi-locus Wright-Fisher process relies on a system of recursions which describe the evolution of neutral sites conditional on the presence of a linked selected site (see Methods). The process of generating those recursions has been automated [46] to handle more general scenarios including population structure and interaction between multiple selected sites. Our model could therefore be extended to handle these more complex scenarios at the expense of (potentially significantly) greater computational effort and data requirements.

For datasets consisting of a small number of time intervals, or which are sampled at low coverage, allele frequency trajectories may be very noisy, making it difficult to reliably detect the presence (or absence) of selection. In these cases, it could be useful to decrease the variance of our estimates by including many more segregating sites into the model in hopes of “averaging out” the noise. The quadratic time complexity of our method makes this difficult to achieve, but alternatives could be explored. These could include approximating the covariance matrix used in the model by something which is faster to compute, (for example, using the Matérn covariance function), or using an ensemble approach whereby a large number of small models are fit simultaneously to the same putative selected site and at various linked neutral sites.

## Methods

Our model (3) posits that the population-level allele frequency array **X** ≡ (*X*_*ijk*_) ∊ [0, 1]^*T*×*L*×*R*^ is conditionally a multidimensional Gaussian random variable. In order to specify such a model, we therefore need to be able to compute the marginal first-order moments 𝔼*X*_*ijk*_, along with the marginal second-order moments 𝔼(*X*_*ijk*_, *X*_*uvw*_), for all times *t*_*i*_, *t*_*u*_ ∊ {*t*_1_, …, *t*_*T*_}, loci *j*, *v* ∊ {1, …, *L*}, and replicates *k*, *w* ∊ {1, …, *R*}. (Since the replicates are assumed to be independent and identically distributed, we suppress the dependence on index *k* for the remainder of this section.)

Below we describe rigorously how to compute the needed moments. First let us give some intuition. The first- and second-order moments described above involve either one or two loci. It is intuitive, and correct in the case of neutrality, that these moments can be computed accurately by studying simpler one- and two-locus Wright-Fisher models, for which computations are significantly easier than when studying the behavior of all *L* loci in the model jointly. (In the non-neutral case a slightly more delicate analysis is required, which we describe below.) Thus we have reduced the difficult problem of determining the joint distribution of *all* the random variables comprised by **X**, to a simpler problem involving the computation of moments in relatively simple and well-understood Wright-Fisher models.

We now make this argument more precise. Recall that **X** consists of marginal allele counts obtained from a population which is assumed to undergo Wright-Fisher random mating. Let us define this process more rigorously. The *L*-locus, biallelic Wright-Fisher process is defined to be the discrete-time Markov process ${\text{Z}}_{t}=(Z_{t}^{\left(1\right)},\ldots ,Z_{t}^{\left(2^{L}\right)})∊\Delta_{2^{L}-1}$, for *t* = 1, 2, …, where

$$\begin{array}{c} \Delta_{m-1}=\left\{\left(y_{1},\ldots ,y_{m}\right)∊{\left[0,1\right]}^{m}:y_{1}+\cdots +y_{m}=1,\;y_{i}\geq 0\;\forall \;i\right\} \end{array}$$

denotes an (*m* − 1)-dimensional simplex. The 2^*L*^ different entries of **Z**_*t*_ correspond to distinct haplotypes. For example, in a two-locus model with alleles *A*_0_ and *A*_1_ at each locus, **Z**_*t*_ is a 4-tuple with the entries corresponding to the population-wide fraction of *A*_1_ *A*_1_, *A*_1_ *A*_0_, *A*_0_ *A*_1_, and *A*_0_ *A*_0_ haplotypes.

Corresponding to the process **Z**_*t*_ is the *L*-dimensional marginal process $X_{t}=(X_{t}^{\left(1\right)},\ldots ,X_{t}^{\left(L\right)})∊{\left[0,1\right]}^{L}$ in which $X_{t}^{\left(j\right)}$ denotes the population frequency of the *A*_1_ allele at locus *j* and time *t*. Thus, in the above two-locus example, if **Z**_*t*_ = (0.1, 0.2, 0.3, 0.4) then **X**_*t*_ = (0.3, 0.4) gives the population-wide marginal frequencies of the *A*_1_ alleles. It is this marginal process which we observe in a pooled sequencing experiment.

Since each entry of **X**_*t*_ is a linear combination of the entries of **Z**_*t*_, it suffices to compute moments of the form $𝔼Z_{t}^{\left(\ell \right)}$ and $\text{cov}(Z_{t}^{\left(\ell \right)},Z_{u}^{\left(m\right)})$ for arbitrary times *t*, *u* and loci ℓ, *m*. As described above, we assume that either zero or one of the *L* loci considered in the model is under selection. We will carry out this computation separately for each of these two cases. Under the assumption that all sites are neutral, we derive an analytic approximation to the mean and covariance of the vector of **Z**_*t*_. The other case we consider is one in which one site is under selection while the rest are neutral. The hitchhiking effect will disturb the mean and variance of nearby linked sites away from what they would be under neutrality. In this case, a different approximation is necessary, which we describe in detail below.

### Neutral case

As described above, in the case of neutrality it suffices to consider covariances between pairs of sites in a two-locus haploid model. The one-generation transition function of the neutral two-locus Wright-Fisher model with recombination fraction *r* is

$$\begin{array}{cc} f & :\Delta_{3}\to \Delta_{3}\\ Z_{t} & \mapsto Z_{t}+rC_{t}\epsilon \end{array}$$ (8)

where **ϵ** ≡ (−1, 1, 1, −1) and $C_{t}\equiv Z_{t}^{\left(1\right)}Z_{t}^{\left(4\right)}-Z_{t}^{\left(2\right)}Z_{t}^{\left(3\right)}$ is the linkage disequilibrium at time *t*. Thus, conditional on **Z**_*t*_ we have that 2*N* × **Z**_*t*+1_ is multinomially distributed according to *f*(**Z**_*t*_):

$$\begin{array}{c} 2NZ_{t+1}|Z_{t}∼\mathrm{Multinomial}\left(2N,f\left(Z_{t}\right)\right). \end{array}$$ (9)

(Note that the multinomial distribution which arises in this equation is due to the random sampling of gametes to form generation *t* + 1, and is different from the binomial sampling scheme described earlier (equation 2) which was resulted from sampling biallelic sites using sequencer.)

Using equation (9), we can derive an accurate approximation to the evolution of the covariance of the **Z**_*t*_ process. In what follows we let *π* = (*z* ^(1)^, *z* ^(2)^, *z* ^(3)^, *z* ^(4)^) and *c*_0_ = *z* ^(1)^ *z* ^(4)^ − *z* ^(2)^ *z* ^(3)^ denote the initial distribution and linkage disequilibrium of the Wright-Fisher process under consideration.

**Lemma 1**. *To order* $O(r+\frac{1}{2N})$,

$$\begin{array}{cc} {𝔼}_{\pi }Z_{t}^{\left(i\right)} & =z^{\left(i\right)}+\epsilon_{i}trc_{0}\;\left(1-\frac{t-1}{4N}\right)\\ {𝔼}_{\pi }\left(rZ_{t}^{\left(i\right)}Z_{t}^{\left(j\right)}\right) & =\frac{r}{2N}\;\left[z^{\left(i\right)}z^{\left(j\right)}\left(2N-t\right)+tz^{\left(i\right)}1\left\{i=j\right\}\right]\\ {𝔼}_{\pi }\left(rZ_{t}^{\left(i\right)}C_{t}\right) & =\frac{r}{2N}\;\left[z^{\left(i\right)}c_{0}\left(2N-3t\right)+\frac{t}{2}\left(\left(1-\epsilon_{i}\right)z^{\left(1\right)}z^{\left(4\right)}-\left(1+\epsilon_{i}\right)z^{\left(2\right)}z^{\left(3\right)}\right)\right]. \end{array}$$

**Corollary 2**. *To order* $O(r+\frac{1}{2N})$,

$$\begin{array}{c} {𝔼}_{\pi }\left(Z_{t}^{\left(i\right)}Z_{t}^{\left(j\right)}\right)=z^{\left(i\right)}z^{\left(j\right)}+\epsilon_{i}\epsilon_{j}trc_{0}\left(\epsilon_{i}z^{\left(i\right)}+\epsilon_{j}z^{\left(j\right)}\right)+\frac{t}{2N}\left(-z^{\left(i\right)}z^{\left(j\right)}1_{\left\{i\neq j\right\}}+z^{\left(i\right)}\left(1-z^{\left(j\right)}\right)1_{\left\{i=j\right\}}\right)\\ \frac{rt}{2N}\;\{\frac{1}{2}\left(t+1-|\epsilon_{i}-\epsilon_{j}|\right)\;\left(z^{\left(1\right)}z^{\left(4\right)}+z^{\left(2\right)}z^{\left(3\right)}\right)-\epsilon_{i}\epsilon_{j}c_{0}\left(2t-1\right)\left(\epsilon_{i}z^{\left(i\right)}+\epsilon_{j}z^{\left(j\right)}\right)-\\ \frac{1}{8}\left|\epsilon_{i}+\epsilon_{j}\right|\;\left[c_{0}\left(\epsilon_{i}+\epsilon_{j}\right)\left(t+1\right)1_{\left\{i\neq j\right\}}+4t\left(\left(\epsilon_{i}+1\right)z^{\left(2\right)}z^{\left(3\right)}+\left(1-\epsilon_{i}\right)z^{\left(1\right)}z^{\left(4\right)}\right)\right]\}. \end{array}$$

Proofs of the above results are given in S1 Text. These results can be combined to give an $O(r+\frac{1}{2N})$ approximation to the within-generation covariance ${\text{cov}}_{\pi }(Z_{t}^{\left(i\right)},Z_{t}^{\left(j\right)})$. Using the same approach, we can also approximate the covariance between generations. Indeed, by Lemma 1 and the Markov property,

$$\begin{array}{c} {𝔼}_{\pi }\left(Z_{t+u}^{\left(i\right)}|Z_{t}\right)={𝔼}_{Z_{t}}\left(Z_{u}^{\left(i\right)}\right)=Z_{t}^{\left(i\right)}+\epsilon_{i}urC_{t}\;\left(1-\frac{u-1}{4N}\right). \end{array}$$

Hence,

$$\begin{array}{c} {𝔼}_{\pi }\left(Z_{t+u}^{\left(i\right)},Z_{t}^{\left(j\right)}\right)={𝔼}_{\pi }\left[Z_{t}^{\left(i\right)}Z_{t}^{\left(j\right)}+\epsilon_{i}urZ_{t}^{\left(j\right)}C_{t}\;\left(1-\frac{u-1}{4N}\right)\right] \end{array}$$

and each of the expectations on the right-hand side is given to order $O(r+\frac{1}{2N})$ by the preceding results.

*Remark*. The constants subsumed in the $O(r^{2}+\frac{1}{{\left(2N\right)}^{2}})$ terms in the above expressions increase as *t* increases; in particular, we would not expect the approximation to be accurate if *tr* ∊ *O*(1). For our application typically *t* ≪ 1/*r*.

### Non-neutral case

Computations in the non-neutral case are more involved because the transition operator *f*(**Z**_*t*_) is a rational function of its arguments. This results in moments of **Z**_*t*+1_ depending on *all* moments of **Z**_*t*_. To illustrate the issues involved, consider first the simplest possible example of a one-locus Wright-Fisher model with diploid selection and no mutation [39]. The relative fitnesses of *A*_0_/*A*_0_ and *A*_1_/*A*_1_ homozygote genotypes are given by 1 and 1 + *s*, respectively, whereas the relative fitness of the *A*_0_/*A*_1_ heterozygote is 1 + *hs*. The frequency of the *A*_1_ allele at time *t* is denoted *X*_*t*_. Conditional on *X*_*t*_, 2*N*×*X*_*t*+1_ has a binomial distribution with 2*N* trials and success parameter *f*(*X*_*t*_), where

$$\begin{array}{c} f\left(x\right)=x+\frac{s[h+(1-2h)x]x(1-x)}{1+sx[2h+(1-2h)x]}. \end{array}$$ (10)

We cannot apply the method described in the preceding subsection due to the appearance of *x* in the denominator of (10). Hence, a different form of approximation is required. First, we formally decompose *X*_*t*_ as $X_{t}={\bar{X}}_{t}+\delta X_{t}$, where ${\bar{X}}_{t}=f({\bar{X}}_{t-1})$ equals the deterministic trajectory that would be followed by *X*_*t*_ in the infinite-population limit, and *δX*_*t*_ is a random disturbance away from the deterministic path due to genetic drift. Next, we expand 𝔼(*X*_*t*_) in a Taylor series about this deterministic path:

$$\begin{array}{cc} 𝔼(X_{t}) & =𝔼(f\left(X_{t-1}\right))\\ & =𝔼(f\left({\bar{X}}_{t-1}+\delta X_{t-1}\right))\\ & \approx f\left({\bar{X}}_{t-1}\right)+{\left.\frac{df}{dx}\right|}_{{\bar{X}}_{t-1}}\times 𝔼\left(\delta X_{t-1}\right)+{\left.\frac{1}{2}\frac{d^{2}f}{dx^{2}}\right|}_{{\bar{X}}_{t-1}}\times 𝔼\left[{\left(\delta X_{t-1}\right)}^{2}\right]. \end{array}$$

This yields a recursion for computing 𝔼(*X*_*t*_) in terms of moments of the disturbance term in the preceding time period, 𝔼[(*δX*_*t*−1_)^*u*^], *u* = 1, 2. Since also

$$\begin{array}{c} 𝔼\left(X_{t}\right)={\bar{X}}_{t}+𝔼\left(\delta X_{t}\right)=f\left({\bar{X}}_{t-1}\right)+𝔼\left(\delta X_{t}\right), \end{array}$$

these terms themselves obey the recursion

$$\begin{array}{c} 𝔼\left(\delta X_{t}\right)\approx {\left.\frac{df}{dx}\right|}_{{\bar{X}}_{t-1}}\times 𝔼\left(\delta X_{t-1}\right)+{\left.\frac{1}{2}\frac{d^{2}f}{dx^{2}}\right|}_{{\bar{X}}_{t-1}}\times 𝔼\left[{\left(\delta X_{t-1}\right)}^{2}\right] \end{array}$$

which is a recursion for computing 𝔼(*δX*_*t*_) in terms of the moments of *δX*_*t*−1_. Inductively assuming that we can compute 𝔼[(*δX*_*t*_)^*u*^] for *u* = 1, 2, this enables us to compute 𝔼(*X*_*t*_) and var(*X*_*t*_) = var(*δX*_*t*_).

This approach was previously employed by Barton *et al*. [47] to obtain order *O*(1/*N*) approximations to these moments. Here we have used the same idea but automated the symbolic algebra and code generation needed to generate the recursions to higher orders of accuracy.

### Multi-locus case

The above idea can be extended to multiple loci in a straightforward manner. (As we describe in the next subsection, we only require models of size up to *L* = 3 for our purposes, but we state it in full generality here.) Recall ${\text{Z}}_{t}=(Z_{t}^{\left(1\right)},\ldots ,Z_{t}^{\left(2^{L}\right)})∊\Delta_{2^{L}-1}$. Conditional on **Z**_*t*_, the vector 2*N* × **Z**_*t*+1_ is multinomially distributed with success probabilities *f*(**Z**_*t*_). The form of *f*:Δ_2^*L*^−1_ → Δ_2^*L*^−1_ varies according to the underlying model; we describe our choice of *f* in the following subsection.

As in the one-locus case, write $Z_{t}^{\left(i\right)}={\bar{Z}}_{t}^{\left(i\right)}+\delta Z_{t}^{\left(i\right)}$ where ${\bar{Z}}_{t}^{\left(i\right)}$ is the deterministic trajectory which would be followed by $Z_{t}^{\left(i\right)}$ in the infinite-population limit, and $\delta Z_{t}^{\left(i\right)}$ is a random disturbance. (Note that in general, $𝔼(\delta Z_{t}^{\left(i\right)})\neq 0$ for *t* > 1.) For *u*, *v* non-negative integers, we have

$$\begin{array}{cc} 𝔼\left[{\left(Z_{t}^{\left(i\right)}\right)}^{u}{\left(Z_{t}^{\left(j\right)}\right)}^{v}\right] & =\;𝔼\left[{\left({\bar{Z}}_{t}^{\left(i\right)}+\delta Z_{t}^{\left(i\right)}\right)}^{u}{\left({\bar{Z}}_{t}^{\left(j\right)}+\delta Z_{t}^{\left(j\right)}\right)}^{v}\right]\\ & =\;𝔼\left[{\left({\bar{Z}}_{t}^{\left(i\right)}+\delta Z_{t}^{\left(i\right)}\right)}^{u}{\left({\bar{Z}}_{t}^{\left(j\right)}+\delta Z_{t}^{\left(j\right)}\right)}^{v}-{\left(\delta Z_{t}^{\left(i\right)}\right)}^{u}{\left(\delta Z_{t}^{\left(j\right)}\right)}^{v}\right]+\\ & \;\;𝔼\left[{\left(\delta Z_{t}^{\left(i\right)}\right)}^{u}{\left(\delta Z_{t}^{\left(j\right)}\right)}^{v}\right]. \end{array}$$ (11)

From the conditional distribution 2*N* **Z**_*t*_∣**Z**_*t*−1_ ∼ 𝓑(2*N*, *f*(**Z**_*t*−1_)), we have

$$\begin{array}{c} {\left(2N\right)}^{u+v}\cdot 𝔼\left[{\left(Z_{t}^{\left(i\right)}\right)}^{u}{\left(Z_{t}^{\left(j\right)}\right)}^{v}|Z_{t-1}\right]=g_{ij}\left(f\left(Z_{t-1}\right)\right)=g_{ij}\left(f\left({\bar{Z}}_{t-1}+\delta Z_{t-1}\right)\right), \end{array}$$

where *g*_*ij*_(*z* ^(1)^, …, *z* ^(2^*L*^)^) is a polynomial in *z* ^(1)^, …, *z* ^(2^*L*^)^ which can be computed using the moment generating function of the multinomial distribution. By performing a Taylor expansion of *h*_*ij*_ ≡ *g*_*ij*_ ∘ *f* about the deterministic path ${\bar{\text{Z}}}_{t-1}$ and taking expectations, we get another formula for $𝔼[{\left(Z_{t}^{\left(i\right)}\right)}^{u}{\left(Z_{t}^{\left(j\right)}\right)}^{v}]$ in terms of moments of *δ* **Z**_*t*−1_:

$$\begin{array}{c} 𝔼\left[{\left(Z_{t}^{\left(i\right)}\right)}^{u}{\left(Z_{t}^{\left(j\right)}\right)}^{v}\right]\approx h_{ij}\left({\bar{Z}}_{t-1}\right)+\sum_{l}{\left.\frac{\partial h_{ij}}{\partial z^{\left(l\right)}}\right|}_{{\bar{Z}}_{t-1}}𝔼\left(\delta Z_{t-1}^{\left(l\right)}\right)+\frac{1}{2}\sum_{l,m}{\left.\frac{\partial h_{ij}}{\partial z^{\left(l\right)}\partial z^{\left(m\right)}}\right|}_{{\bar{Z}}_{t-1}}𝔼\left(\delta Z_{t-1}^{\left(l\right)}\delta Z_{t-1}^{\left(m\right)}\right). \end{array}$$ (12)

For *u* + *v* ≤ 2, comparing (11) and (12) yields a recursion for computing $𝔼\left[{\left(\delta Z_{t}^{\left(i\right)}\right)}^{u}{\left(\delta Z_{t}^{\left(j\right)}\right)}^{v}\right]$ in terms of moments of *δ* **Z**_*t*_ of total degree strictly less than *u* + *v*, and moments *δ* **Z**_*t*−1_ of total degree at most *u* + *v*. The latter feature is important for computation because it implies that we only need to compute a bounded number of terms in each recursive step, which would not be the case if we had instead expanded the function *h*_*ij*_(⋅) about zero with respect to model parameters (for example, selection or mutation).

The recursive nature of the above algorithm lends itself to computing moments of the form $\text{cov}(\delta Z_{t+m}^{\left(i\right)},\delta Z_{t}^{\left(j\right)})$. Stopping the recursion *m* time steps into the past, we obtain an expression of the form $𝔼(\delta Z_{t+m}^{\left(i\right)}|\delta {\text{Z}}_{t})={𝑝}_{im}(\delta {\text{Z}}_{t}),$ where 𝑝_*im*_(*z* ^(1)^, …, *z* ^(2^*L*^)^) is a polynomial. Hence,

$$\begin{array}{c} 𝔼\left(\delta Z_{t+m}^{\left(i\right)}\delta Z_{t}^{\left(j\right)}\right)=𝔼\left(\delta Z_{t}^{\left(j\right)}{𝑝}_{im}\left(\delta Z_{t}\right)\right) \end{array}$$

is again a recursion involving moments of *δ* **Z**_*t*_ which can be solved using the techniques described above.

### Moment calculation with a linked selected site

When selection is acting on a nearby linked site, some additional care is needed in computing the first- and second-order moments for neutral sites. For example, the hitchhiking effect will cause these moments to be different from they would be in the absence of linked selection. Consider a three-locus model with **X**_*t*_ = (*X*_*t*, 1_, *X*_*t*, 2_, *X*_*t*, 3_), where *X*_*t*, *j*_ denotes the marginal allele frequency at time *t* at locus *j*. Suppose the site corresponding to *X*_*t*,1_ is under positive selection, and the remaining sites are neutral and under positive LD with the selected site. Computing 𝔼*X*_*t*,2_ using a one-locus neutral model as described above will produce an underestimate since linkage with site 1 will cause site 2 to rise in frequency faster than what is expected under neutrality. A similar effect can be seen when computing 𝔼(*X*_*t*,2_ *X*_*t*,3_).

To capture this effect it is necessary to condition on the presence of a linked selected site when performing the moment calculations discussed earlier for neutral sites. To carry this out we utilize a three-locus model which describes the evolution of two neutral and one linked selected site over time. This model was derived by Stephan *et al*. [48] using the general framework of Kirkpatrick *et al*. [46]. In the notation of the preceding subsection, we let *L* = 3 and obtain the transition function *f* using the system of recursions presented in equations (1)–(11) of [48]. This system can then be expanded in terms of the random disturbance *δ* **Z**_*t*_ to yield the system of recursions (11) and (12). The differentiation steps needed to perform the expansion involve a very large number of terms, and are too complex to perform by hand. Instead, we automated these computations using the symbolic algebra package Maple. Code to automatically generate these recursions is included in the source code accompanying this paper.

### Simulation

Our procedure for simulating an E&R experiment was the following. To generate realistic patterns of standing variation, a set of *F* founder lines was sampled from the coalescent with recombination using the program ms [49]. (The exact ms command-line used for each simulation was: ms <*F*> 1 -t <4*μLN*_*e*_> -r <4*N*_*e*_(*L* − 1)*r*> <*L*>, where the variables in angled brackets are computed using the values described in the text.) Recombination and mutation rates and the effective population size were set to biologically plausible values for *D. melanogaster*, a common model organism used in E&R studies (*r* = 2 × 10^−8^/bp/gen, *μ*/2 = 10^−9^/bp/gen, *N* = 10^6^) [50]. Each founder line was cloned 2*N*/*F* times to generate an initial diploid population of size *N*. This replication step is intended to mimic the practice using of (nearly-)homozygous recombinant inbred founder lines to initialize an E&R experiment. Next, the experimental population of size *N* was simulated forward in time using the discrete-time simulator simuPOP [51]. Finally, alleles were sampled binomially and independently at each locus and time point to simulate next-generation sequencing. Parameters for the forward simulation and sampling were varied from scenario to scenario as described in the main text. The output of the simulation consisted of the haplotypes of the initial founder lines and the frequency of each segregating site (potentially after sampling) at each time point. All simulations were performed on a machine with 2 × 2.5 GHz AMD Opteron 6380 processors (32 cores total) and 256 GB of memory.

### Analysis of real data

In our model, we used an effective population size of 200, as previously estimated for the E&R data we considered [25]. To prevent our estimates from becoming confounded by the action of genetic drift, we restricted our analysis to only those sites which were segregating at intermediate frequencies throughout the experiment. Specifically, we only considered sites which were segregating at frequencies in the interval [0.1, 0.9] for all generations and replicates. A total of 414, 049 sites remained after filtering.

First, we computed the one-locus likelihood-ratio statistic at each of the 414, 049 sites, comparing the fitted model to the null (neutral) model. Then, we partitioned the genome into non-overlapping windows of a fixed size (we considered various window sizes, including 5 kb, 10 kb, 50 kb, 100 kb, 200 kb, 500 kb, and 1 Mb) and computed the average one-locus LR statistic over the SNPs in each window. By visually inspecting plots of these quantities, we identified regions of the genome which were enriched for SNPs that potentially behaved non-neutrally.

For each region identified, a multi-locus model was then estimated for each segregating site within the region. Specifically, we fit a model in which each site in the region was posited to be under selection, and the trajectories of linked neutral sites were affected due to hitchhiking. To choose which linked neutral to include in the model, we identified SNPs which were segregating at multiples of approximately 250 kb from the midpoint of the region. For example, to analyze the region 6.6–6.7 Mb on chromosome 3L using a five-site model, we first fixed four SNPs segregating at intermediate frequencies near positions 6.15 Mb, 6.4 Mb, 6.9 Mb and 7.15 Mb. For each site between 6.6 Mb and 6.7 Mb, we then estimated the strength of selection *s* using the five-locus model containing the selected site plus the four fixed neutral sites.

## Supporting Information

S1 Text Proofs of Lemma 1 and Corollary 2. (PDF)

S1 Fig Empirical coverage distribution. Empirical coverage $\hat{C}$ observed in a real E&R experiment of *Drosophila melanogaster* [25, 36]. The distribution has high average coverage $\left(E\hat{C}=84.2\right)$ but with a heavy left-tail which results in low to no coverage for a small fraction of the sites.
(PDF)

S2 Fig Pooled coverage distribution. Coverage distribution for pooled sequencing experiments. Sequencing was performed in generations 15, 37 and 59, for three replicates labeled 1, 4 and 5.
(PDF)

S3 Fig Comparison of the one-locus and multi-locus models applied to a real E&R experiment. Shown here are the likelihood-ratio statistics for a 800 kb region of *D. melanogaster* chromosome arm 3L. Every multi-locus model yielded a distinctive peak (shown in red) near 18.205 Mb of chromosome arm 3L, while the one-locus model did not single out any particular SNPs in the region.
(PDF)

S4 Fig Empirical cumulative distributions of the LR statistic for each multi-locus model. The 99th percentile for the 3-, 5-, and 7-locus models are 6.883, 7.330, and 8.257, respectively.
(PDF)

S5 Fig Allele frequency trajectories of the thirteen top SNPs identified by our multi-locus analysis. Each SNP has three trajectories corresponding to the three replicate experiments. The initial frequency at generation 0 was estimated from pooled sequencing data for the base population. Note that all thirteen SNPs generally display an upward trend over the time course of the experiment.
(PDF)

S1 Table Results of localization procedure, intermediate MAF. This table displays the same results as Table 1, except that here we only consider those simulations in which the selected site was segregating at a frequency of at least 0.1 in the initial generation. Note that increasing *F* improves the ability to localize the selected site for *s* ∊ {0.02, 0.05}; for strong selection (*s* = 0.1), essentially all cases of *F* performed equally well.
(PDF)

S1 Code Source code implementing the method described in this paper. (ZIP)
