# Supplementary material for: Multi-locus Analysis of Genomic Time Series Data from Experimental Evolution
Source: PLoS Genet. 2015 Apr 7;11(4):e1005069. doi: 10.1371/journal.pgen.1005069 (PMC4388667; doi:10.1371/journal.pgen.1005069)
Supplement: S1 Text — (PDF) [file pgen.1005069.s001.pdf]

Supporting Information

Multi-locus analysis of genomic time series data from experimental evolution

Jonathan Terhorst[1], Christian Schlötterer[4], Yun S. Song[1,2,3,*]

[1] Department of Statistics, [2] Computer Science Division, [3] Department of Integrative Biology, University of California, Berkeley, CA, USA
[4] Institut für Populationsgenetik, Vetmeduni Vienna, Vienna, Austria

* Corresponding author e-mail: yss@cs.berkeley.edu

December 29, 2014

Text S1

Here we provide proofs of the theoretical results mentioned in the **Methods** section of the main text.

Lemma 1. *To order* $O(r + \frac{1}{2N})$,

$$\begin{aligned}
\mathbb{E}_\pi Z_t^{(i)} &= z^{(i)} + \epsilon_i t r c_0 \left(1 - \frac{t-1}{4N}\right) \\
\mathbb{E}_\pi (r Z_t^{(i)} Z_t^{(j)}) &= \frac{r}{2N}\left[z^{(i)} z^{(j)} (2N - t) + t z^{(i)} \mathbf{1}\{i = j\}\right] \\
\mathbb{E}_\pi (r Z_t^{(i)} C_t) &= \frac{r}{2N}\left[z^{(i)} c_0 \, (2N - 3t) + \frac{t}{2}\left((1-\epsilon_i) z^{(1)} z^{(4)} - (1+\epsilon_i) z^{(2)} z^{(3)}\right)\right].
\end{aligned}$$

Proof of Lemma 1. By direct computation using the moment generating function of the multinomial distribution, we find that

$$\begin{aligned}
\mathbb{E}_\pi (Z_t^{(i)} \mid \mathbf{Z}_{t-1}) &= Z_{t-1}^{(i)} + \epsilon_i r C_{t-1} \\
\mathbb{E}_\pi (r Z_t^{(i)} Z_t^{(j)} \mid \mathbf{Z}_{t-1}) &= r Z_{t-1}^{(i)} Z_{t-1}^{(j)} \left(1 - \frac{1}{N}\right) + Z_{t-1}^{(i)} \mathbf{1}\{i = j\} + O(r^2 + 1/N^2) \\
\mathbb{E}_\pi (r C_t Z_t^{(i)} \mid \mathbf{Z}_{t-1}) &= \frac{r}{N}\Big[Z_{t-1}^{(i)} C_{t-1} (N - 3) \\
&\qquad + \frac{1}{2}\left((1-\epsilon_i) z^{(1)} z^{(4)} - (1+\epsilon_i) z^{(2)} z^{(3)}\right)\Big] + O(r^2 + 1/N^2).
\end{aligned}$$

The results now follow by induction. ☐

Corollary 2. *To order* $O(r + \frac{1}{2N})$,

$$\begin{aligned}
\mathbb{E}_\pi \left(Z_t^{(i)} Z_t^{(j)}\right) &= z^{(i)} z^{(j)} + \epsilon_i \epsilon_j t r c_0 (\epsilon_i z^{(i)} + \epsilon_j z^{(j)}) + \frac{t}{2N}\left(-z^{(i)} z^{(j)} \mathbf{1}_{\{i \neq j\}} + z^{(i)} (1 - z^{(j)}) \mathbf{1}_{\{i = j\}}\right) \\
&\frac{rt}{2N}\Big\{\frac{1}{2}\left(t + 1 - |\epsilon_i - \epsilon_j|\right)\left(z^{(1)} z^{(4)} + z^{(2)} z^{(3)}\right) - \epsilon_i \epsilon_j c_0 (2t - 1)(\epsilon_i z^{(i)} + \epsilon_j z^{(j)}) - \\
&\qquad \frac{1}{8}|\epsilon_i + \epsilon_j|\left[c_0 (\epsilon_i + \epsilon_j)(t+1) \mathbf{1}_{\{i \neq j\}} + 4t\big((\epsilon_i + 1) z^{(2)} z^{(3)} + (1 - \epsilon_i) z^{(1)} z^{(4)}\big)\right]\Big\}.
\end{aligned}$$

Proof of Corollary 2. The formula is obtained by considering the conditional expectation $\mathbb{E}_\pi(Z_t^{(i)} Z_t^{(j)} \mid \mathbf{Z}_{t-1})$, inducting on t and checking cases for i and j. We illustrate the proof for the case $i = j = 1$ and omit the lengthy but routine computations used to check the remaining cases. (A Mathematica notebook which checks all cases is available from the authors upon request.) With $i = j = 1$ we have

$$\mathbb{E}_\pi\left[\left(Z_t^{(1)}\right)^2 \middle| \mathbf{Z}_{t-1}\right] = \frac{1}{N}\left[Z_{t-1}^{(1)}(1 - Z_{t-1}^{(1)}) - rC_{t-1}\left(1 - 2Z_{t-1}^{(1)}\right)\right] \\ - rC_{t-1}Z_{t-1}^{(1)} + O(r^2 + 1/N^2).$$

This yields the claim for $t = 1$. Taking expectation and applying the preceding lemma, we find that

$$\mathbb{E}_\pi\left[\left(Z_t^{(1)}\right)^2\right] = \frac{1}{N}\Big[z_1 - trc_0 + (N-1)\mathbb{E}_\pi\left(Z_{t-1}^{(1)}\right)^2 - \\ 2rz_1\left(c_0(N - 3t + 3) + (t-1)z_4\right)\Big] + O(r^2 + 1/N^2).$$

Applying the inductive hypothesis, we obtain

$$\mathbb{E}_\pi\left[\left(Z_t^{(1)}\right)^2\right] = \frac{1}{N}\Big[z_1 - trc_0 + z_1\left(t - 1 + (n - t + 1)z_1 - (t-1)(t-2)rz_4\right) - \\ (t-1)rc_0\left[\frac{t}{2} + 2\left(n - 2t + 3\right)z_1\right] - z_1^2 + 2trc_0z_1 - \\ 2rz_1\left(c_0(n - 3t + 3) + (t-1)z_4\right)\Big] + O(r^2 + 1/N^2),$$

which agrees with the claim after some simplification. □
