# Supplementary figures and images for: Multi-locus Analysis of Genomic Time Series Data from Experimental Evolution
Source: PLoS Genet. 2015 Apr 7;11(4):e1005069. doi: 10.1371/journal.pgen.1005069 (PMC4388667; doi:10.1371/journal.pgen.1005069)

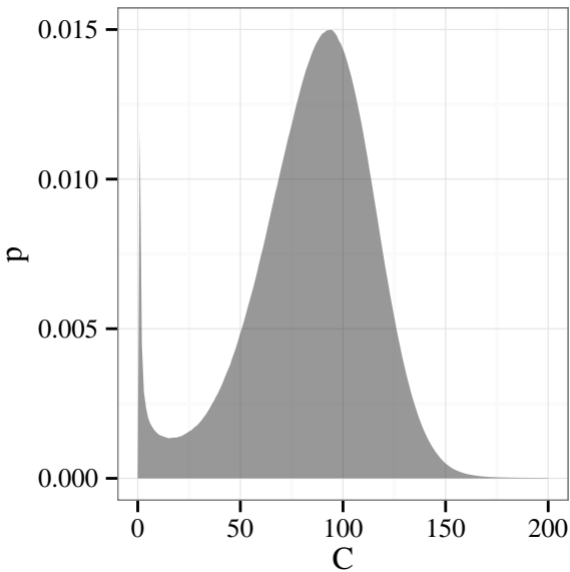

Supplement: S1 Fig — Empirical coverage C^ observed in a real E&R experiment of Drosophila melanogaster [25, 36]. The distribution has high average coverage (EC^=84.2) but with a heavy left-tail which results in low to no coverage for a small fraction of the sites. (PDF) [file pgen.1005069.s002.pdf]

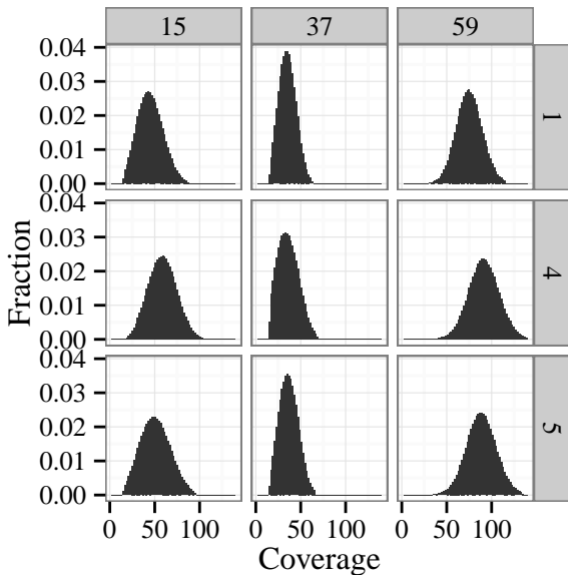

Supplement: S2 Fig — Coverage distribution for pooled sequencing experiments. Sequencing was performed in generations 15, 37 and 59, for three replicates labeled 1, 4 and 5. (PDF) [file pgen.1005069.s003.pdf]

**1-Locus model**

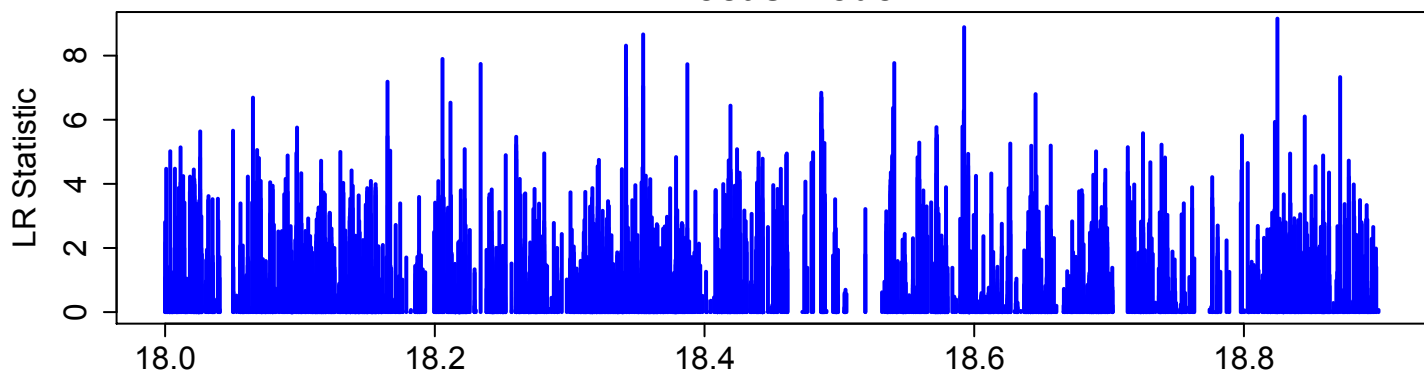

**3-Locus model**

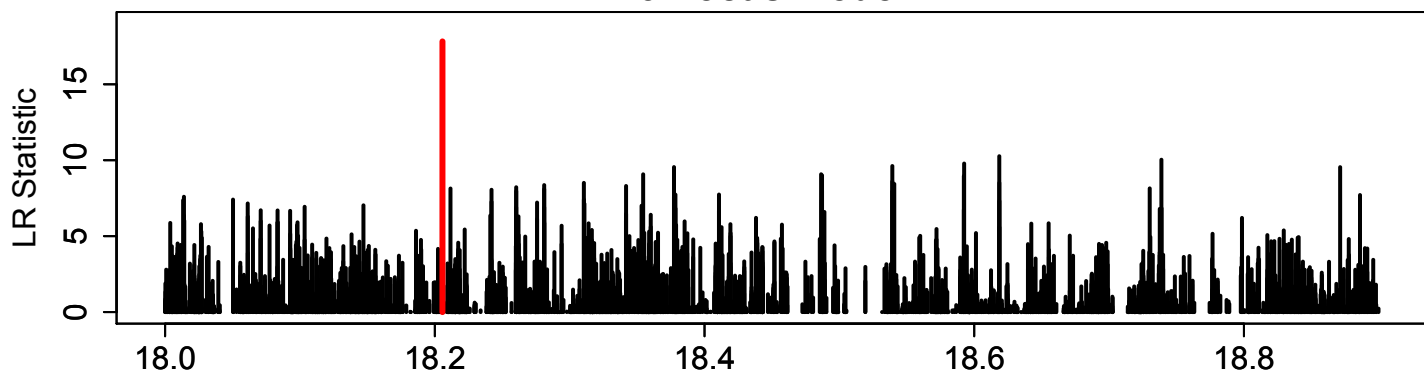

**5-Locus model**

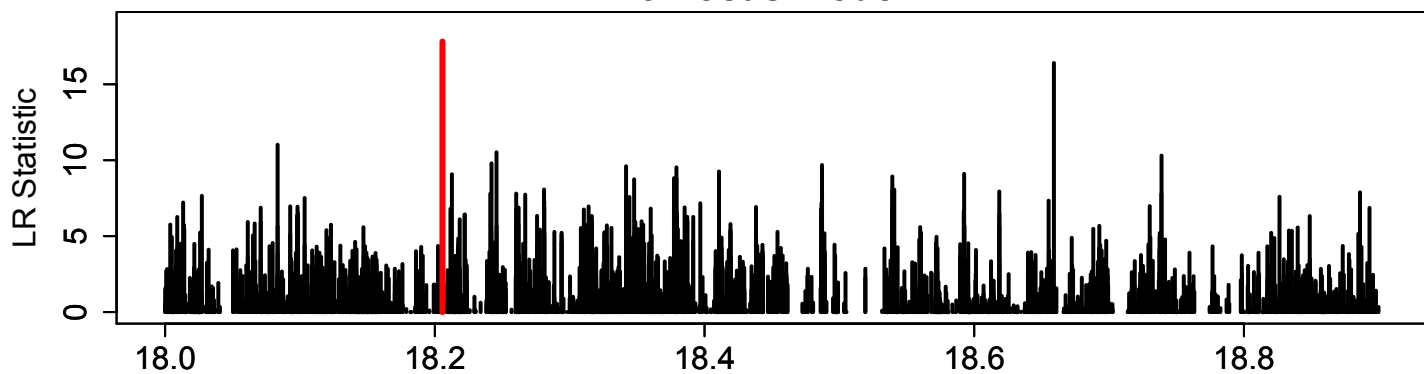

**7-Locus model**

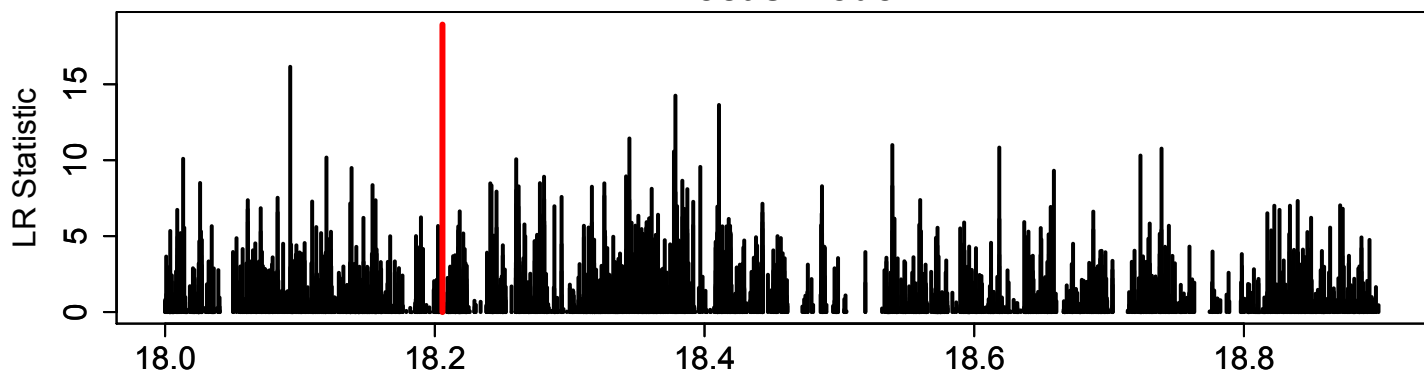

Position (Mb)

Supplement: S3 Fig — Shown here are the likelihood-ratio statistics for a 800 kb region of D. melanogaster chromosome arm 3L. Every multi-locus model yielded a distinctive peak (shown in red) near 18.205 Mb of chromosome arm 3L, while the one-locus model did not single out any particular SNPs in the region. (PDF) [file pgen.1005069.s004.pdf]

**3-locus model**

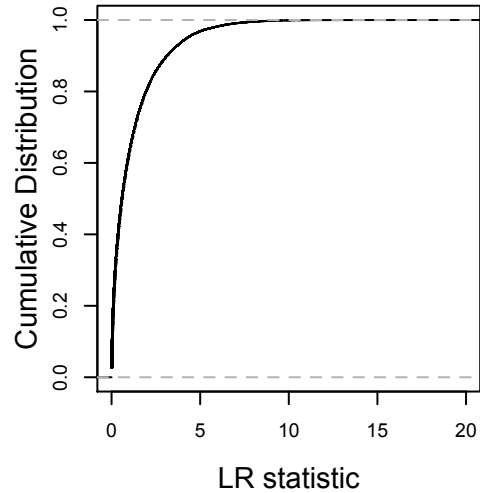

**5-locus model**

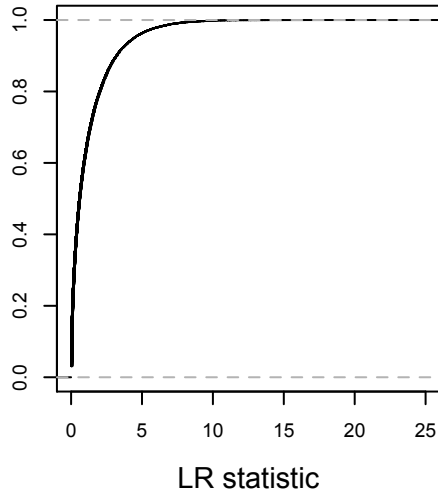

**7-locus model**

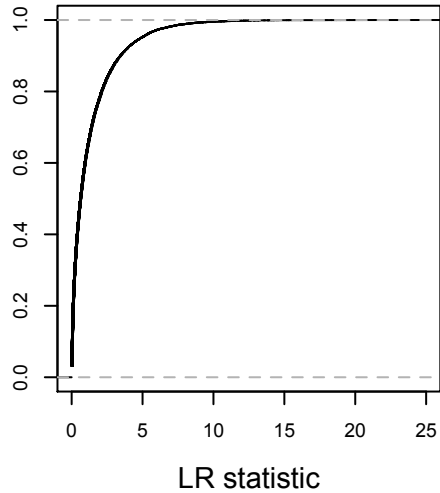

Supplement: S4 Fig — The 99th percentile for the 3-, 5-, and 7-locus models are 6.883, 7.330, and 8.257, respectively. (PDF) [file pgen.1005069.s005.pdf]

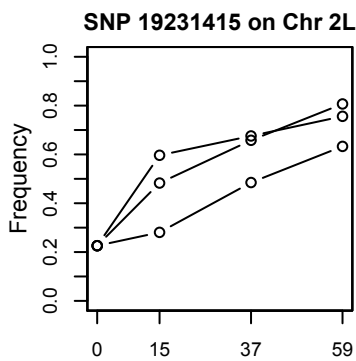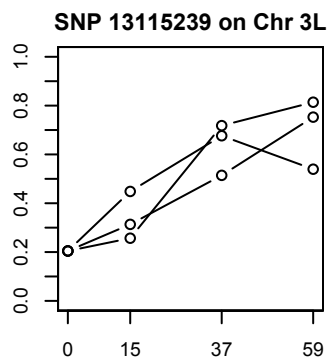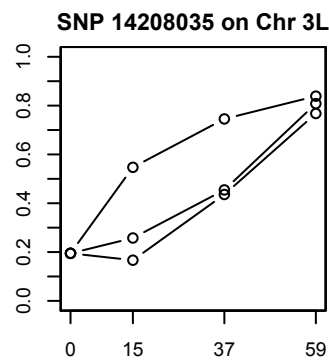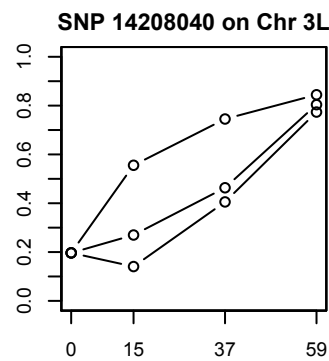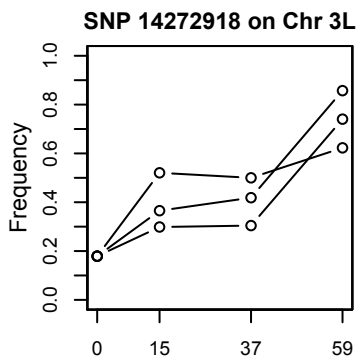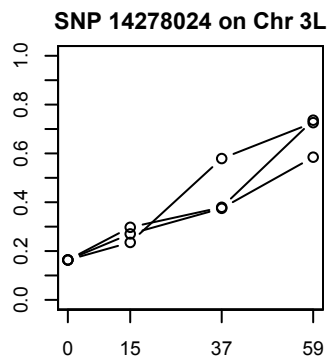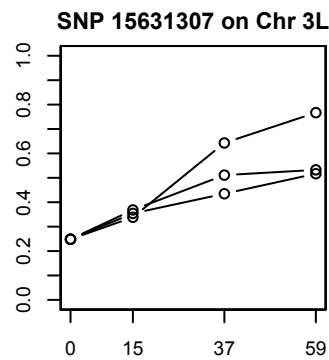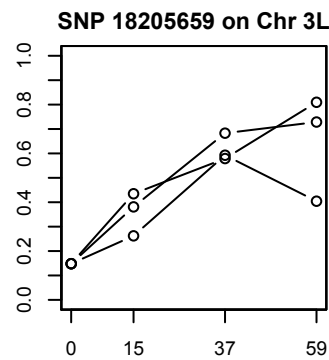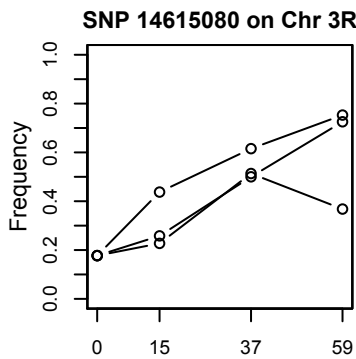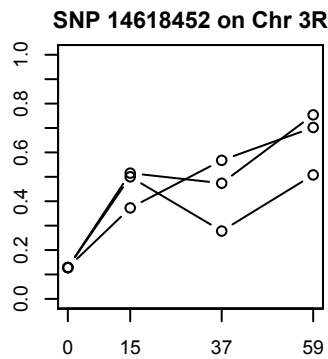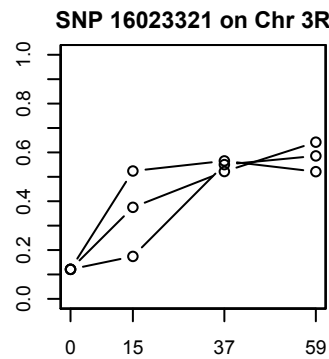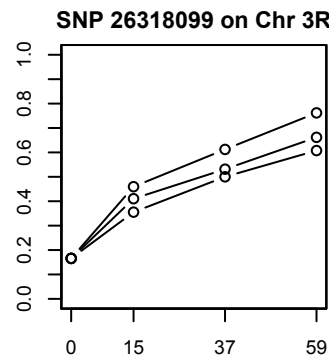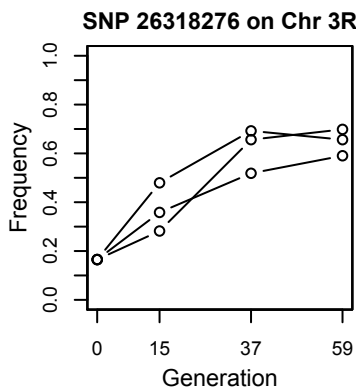

Supplement: S5 Fig — Each SNP has three trajectories corresponding to the three replicate experiments. The initial frequency at generation 0 was estimated from pooled sequencing data for the base population. Note that all thirteen SNPs generally display an upward trend over the time course of the experiment. (PDF) [file pgen.1005069.s006.pdf]
