# Supplementary material for: Multi-locus Analysis of Genomic Time Series Data from Experimental Evolution
Source: PLoS Genet. 2015 Apr 7;11(4):e1005069. doi: 10.1371/journal.pgen.1005069 (PMC4388667; doi:10.1371/journal.pgen.1005069)
Supplement: S1 Table — This table displays the same results as Table 1, except that here we only consider those simulations in which the selected site was segregating at a frequency of at least 0.1 in the initial generation. Note that increasing F improves the ability to localize the selected site for s ∊ {0.02, 0.05}; for strong selection (s = 0.1), essentially all cases of F performed equally well. (PDF) [file pgen.1005069.s007.pdf]

| $s$ | $F$ | Distance | | | | | Rank | | | | | $\mathbb{E}(\#\text{SS})$ |
|---|---|---|---|---|---|---|---|---|---|---|---|---|
| | | $q_{.1}$ | $q_{.25}$ | $q_{.5}$ | $q_{.75}$ | $q_{.9}$ | $q_{.1}$ | $q_{.25}$ | $q_{.5}$ | $q_{.75}$ | $q_{.9}$ | |
| 0.02 | 20 | 610 | 8460 | 27420 | 54960 | 68180 | 4 | 12 | 58 | 435 | 753 | 1306 |
| 0.02 | 200 | 0 | 0 | 12150 | 42921 | 65810 | 1 | 1 | 9 | 113 | 946 | 2082 |
| 0.02 | 2000 | 0 | 4720 | 22330 | 46610 | 70841 | 1 | 7 | 51 | 301 | 986 | 2779 |
| 0.05 | 20 | 0 | 0 | 0 | 16050 | 39370 | 1 | 1 | 1 | 4 | 42 | 1311 |
| 0.05 | 200 | 0 | 0 | 0 | 0 | 3500 | 1 | 1 | 1 | 1 | 3 | 2083 |
| 0.05 | 2000 | 0 | 0 | 0 | 0 | 14120 | 1 | 1 | 1 | 1 | 5 | 2776 |
| 0.10 | 20 | 0 | 0 | 0 | 0 | 1340 | 1 | 1 | 1 | 1 | 2 | 1315 |
| 0.10 | 200 | 0 | 0 | 0 | 0 | 0 | 1 | 1 | 1 | 1 | 1 | 2085 |
| 0.10 | 2000 | 0 | 0 | 0 | 0 | 0 | 1 | 1 | 1 | 1 | 1 | 2778 |
